# An Artificial Intelligence Algorithm for Detection of Severe Aortic Stenosis: A Clinical Cohort Study

**DOI:** 10.1016/j.jacadv.2024.101176

**Published:** 2024-09-25

**Authors:** Jordan B. Strom, David Playford, Simon Stewart, Geoff Strange

**Affiliations:** a Cardiovascular Division, Department of Medicine, Beth Israel Deaconess Medical Center, Boston, Massachusetts, USA; b Richard A. and Susan F. Smith Center for Outcomes Research in Cardiology, Beth Israel Deaconess Medical Center, Boston, Massachusetts, USA; c Division of Cardiovascular Medicine, Beth Israel Deaconess Medical Center, Harvard Medical School, Boston, Massachusetts, USA; d Institute of Health Research, The University of Notre Dame Australia, Fremantle, Western Australia, Australia; e School of Medicine, Dentistry & Nursing, University of Glasgow, Glasgow, Scotland; f The University of Sydney, Faculty of Medicine and Health, Sydney, New South Wales, Australia; g Heart Research Institute, University of Sydney, Sydney, New South Wales, Australia; h Department of Cardiology, Royal Prince Alfred Hospital, Sydney, New South Wales, Australia

**Keywords:** artificial intelligence, decision-support, echocardiography, aortic stenosis

## Abstract

**Background:**

Identifying individuals with severe aortic stenosis (AS) at high risk of mortality remains challenging using current clinical imaging methods.

**Objectives:**

The purpose of this study was to evaluate an artificial intelligence decision support algorithm (AI-DSA) to augment the detection of severe AS within a well-resourced health care setting.

**Methods:**

Agnostic to clinical information, an AI-DSA trained to identify echocardiographic phenotype associated with an aortic valve area (AVA)<1 cm^2^ using minimal input data (excluding left ventricular outflow tract measures) was applied to routine transthoracic echocardiograms (TTE) reports from 31,141 U.S. Medicare beneficiaries at an academic medical center (2003-2017).

**Results:**

Performance of AI-DSA to detect the phenotype associated with an AVA<1 cm^2^ was excellent (sensitivity 82.2%, specificity 98.1%, negative predictive value 9.2%, c-statistic = 0.986). In addition to identifying clinical severe AS cases, AI-DSA identified an additional 1,034 (3.3%) individuals with guideline-defined moderate AS but with a similar clinical and TTE phenotype to those with severe AS with low rates of aortic valve replacement (6.6%). Five-year mortality was 75.9% in those with known severe AS, 73.5% in those with a similar phenotype to severe AS, and 44.6% in those without severe AS. The AI-DSA continued to perform well to identify severe AS among those with a depressed left ventricular ejection fraction. Overall rates of aortic valve replacement remained low, even in those with an AVA<1 cm^2^ (21.9%).

**Conclusions:**

Without relying on left ventricular outflow tract measurements, an AI-DSA used echocardiographic reports to reliably identify the phenotype of severe AS. These results suggest possible utility for this AI-DSA to enhance detection of severe AS individuals at risk for adverse outcomes.

Aortic stenosis (AS) is the most common form of valvular heart disease encountered in clinical practice ^1^^,^^2^ associated with excess costs and premature mortality.^3^ Progression to severe AS is near universally fatal without aortic valve replacement (AVR).^4^ However, despite guideline-based indications to refer patients with symptomatic, severe AS to AVR,^5^^,^^6^ nearly one in 3 such individuals are not referred,^1^^,^^7^, ^8^, ^9^, ^10^ despite an increased mortality risk of 2% per week delayed referral.^11^ Furthermore, patients with moderate AS may experience a mortality risk similar to those with severe AS.^12^, ^13^, ^14^ Identifying these individuals remains challenging using current clinical surveillance methods.

In this setting, artificial intelligence (AI) decision support algorithms (AI-DSAs) may help identify individuals with AS at risk for premature mortality.^9^^,^^15^, ^16^, ^17^, ^18^, ^19^ AI-based systems are being increasingly employed in the clinical management of cardiovascular disease to expedite the detection of specific conditions and to identify who might need proactive treatment to improve health outcomes.^9^^,^^15^, ^16^, ^17^ While often displaying levels of performance comparable or superior to clinical assessment, few AI algorithms have been robustly validated in clinical practice, limiting their rollout and effectiveness.^20^

We aimed to externally validate the performance of a pretrained AI-DSA designed to enhance the detection of AS, in a cohort of U.S. Medicare beneficiaries. This AI-DSA, trained and tested on 631,824 individuals from 1.08 million echocardiograms across Australia,^21^ was specifically designed to use a limited set of echocardiographic parameters to identify individuals with the echocardiographic phenotype of severe AS. Since AS prevalence is highest in older individuals,^2^ we aimed to 1) assess the performance of this AI-DSA in an older, diverse North American cohort, representative of individuals routinely encountered in clinical practice, and 2) compare the outcomes and treatment status of those with severe AS identified by AI-DSA with those who have guideline-derived severe AS. We hypothesized that the AI-DSA would identify the severe AS phenotype similarly to that of the derivation cohort.^21^

## Methods

### Study design

We conducted a retrospective cohort study adherent to the REporting of studies Conducted using Observational Routinely-collected health Data (RECORD) ^22^ and Proposed Requirements for Cardiovascular Imaging-Related Machine Learning Evaluation (PRIME) ^23^ checklists. Institutional Review Board approval was granted by the Beth Israel Deaconess Medical Center (BIDMC). Cell values < 11 are not reported as required by Medicare data use agreements to avoid identification of individuals.

### Study cohort

**Figure 1:**
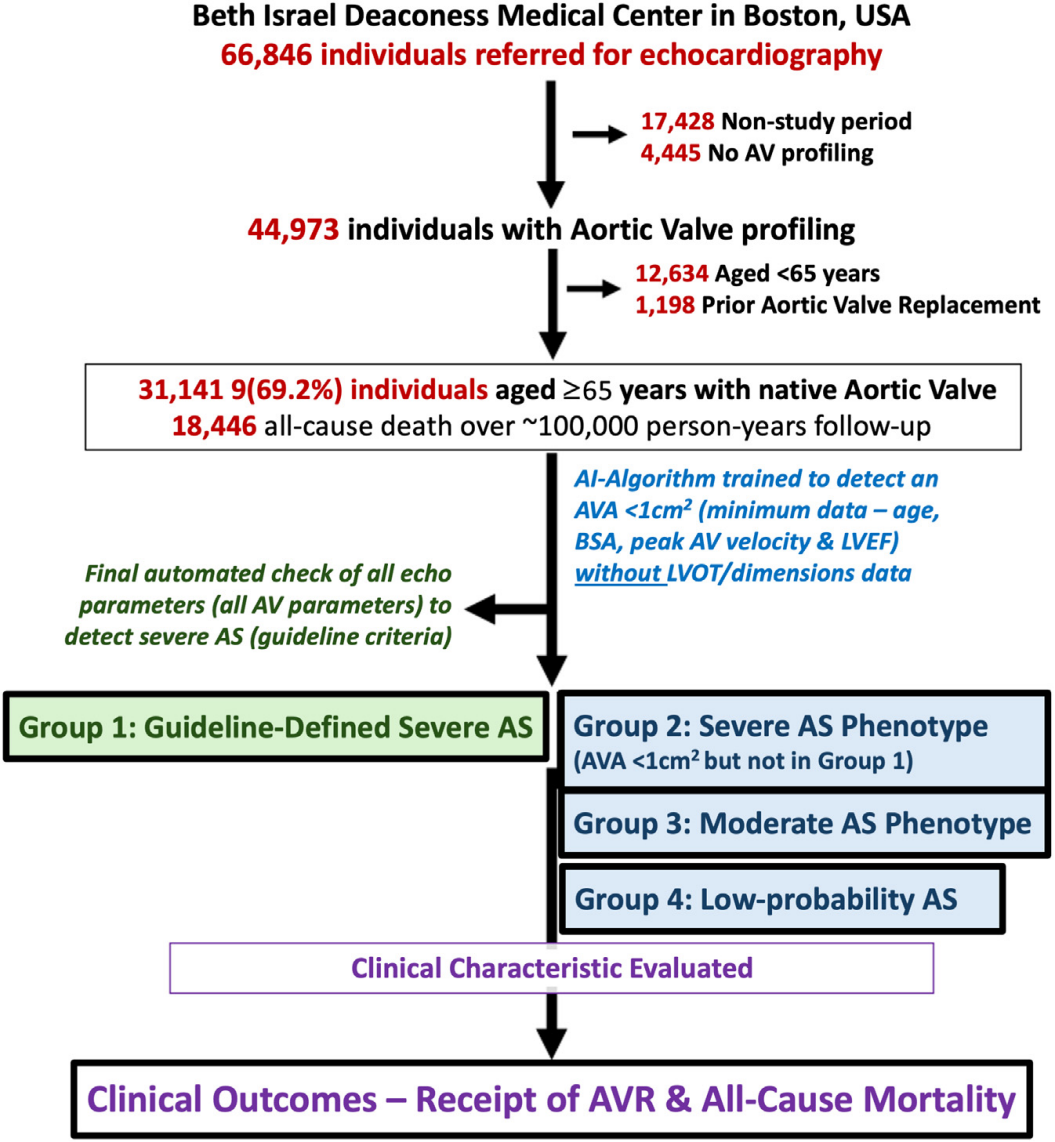
**Study Flow Chart Demonstrating Inclusion and Exclusion Criteria** This graph shows the number of individuals presenting to beth israel deaconess medical center who were included in the current study. Of 66,846 Individuals who underwent transthoracic echocardiography, 17,428 individuals were excluded as being outside the study period, and 4,445 were excluded as they lacked peak aortic velocity information. An additional 12,634 individuals were excluded as being <65 years old at the time of echocardiography and 1,198 individuals were excluded to a prior aortic valve replacement, leaving 31,141 individuals included in the final sample. AI = artificial intelligence; AVA = aortic valve area; AV = aortic valve; AVR = aortic valve replacement; AS = aortic stenosis; BSA = body surface area; LVEF = left ventricular ejection fraction; LVOT = left ventricular outflow tract; SVi = stroke volume index.

The primary cohort comprised 66,846 patients referred for transthoracic echocardiography (TTE) at BIDMC in Boston, Massachusetts, a teaching hospital of Harvard Medical School, from January 1, 2003, to December 31, 2017. Included individuals were ≥65 years at the time of TTE, Medicare-enrolled with linked Medicare fee-for-service claims, had nonmissing aortic peak velocity information (required for model output), with no evidence of prior AVR. A total of 31,141 (69.2%) individuals met inclusion (Figure 1). Consistent with prior methods,^12^ only the last TTE (if multiple TTEs taken) was considered for analysis. The selection of BIDMC as an external validation site was based on the quality and comprehensiveness of the clinical and TTE profiling of predominant hospitalized.^21^

### Covariates

Age, sex, anthropometric profile, and a standard list of left and right heart parameters assessed by TTE were extracted using structured reporting at the time of TTE completion.^24^ Comorbidities using Medicare Chronic Comorbidity Warehouse indicator variables, which use validated algorithms based on complete inpatient/outpatient claims, were used to identify the presence/absence of a clinical condition at the time of the index TTE.^25^ Laboratory values within 3 months prior to the TTE and pharmacologic treatments at the time of TTE were determined using linkage to institutional data sets.^12^ Prior receipt of percutaneous coronary intervention or coronary artery bypass grafting (CABG) was identified via claims in the year preceding the date of TTE.^12^

### Outcomes

Outcomes included all-cause mortality, determined from linkage to Medicare Beneficiary Summary Files, and receipt of AVR, defined using validated claims algorithms.^26^ Time to AVR was defined as the date of TTE to the procedure date of AVR in claims. Comprehensive follow-up was complete for all individuals, regardless of the site of occurrence of AVR, through December 31, 2020.

### Automated quality control

Upon data ingestion, several automatic quality controls were applied.^27^ First, TTE measurements were checked to ensure that values are within humanly possible ranges. Second, computed variables (eg left ventricular mass index) were directly computed from their component parts (eg wall thickness, cavity size, body surface area). Third, if the model encountered a value that represents a clear outlier for the predicted AS phenotype, it would ignore the erroneous measurement.

### Artificial intelligence algorithm

As described in greater detail previously,^17^^,^^21^ the AI-DSA was derived from a multicenter, predominantly outpatient cohort of 631,824 individuals in the National Echocardiographic Database of Australia. After processing, 442,276 (70%) individuals were entered into a Mixture Density Network model to train an AI-DSA to predict the TTE phenotype of an aortic valve area (AVA) < 1.0 cm^2^ derived using the continuity equation.^28^ Mixture Density Networks do not assume that the predicted outputs follow a certain parametric distribution and thus remain robust in circumstances where inaccuracies in measurement (eg left ventricular outflow tract [LVOT] diameter) may create varying parameter distributions. A comprehensive list of 280 routinely collected TTE variables were provided for AI-DSA training (Supplemental Table 1). Mortality status, LVOT diameter, and other variables relevant to AS grading (LVOT velocity and velocity time integral, and AVA) were intentionally excluded from model training to evaluate model resilience to erroneous and sparsely populated data often exist in clinical echocardiograms. The model was trained on markers of the AS phenotype (eg left ventricular dysfunction, hypertrophy) rather than markers of valvular stenosis severity alone. A random 30% subset of variable values were held-back from the model input during each training to evaluate the model’s capacity to predict in sparse data setting. The minimum set of input variables required for the model to generate an output probability included height, weight, sex, left ventricular ejection fraction (LVEF; determined using any technique), and aortic peak velocity. The final model was trained to be general purpose and thus able to compute AS probabilities using any number of input variables, provided that the aforementioned minimal inputs were present. The cumulative incidence function was used to determine that the optimal probability to identify cases with an AVA <1.0 cm^2^ was >0.235 (F1 threshold). Subsequently, the performance of this AI-DSA to detect an AVA <1.0 cm^2^ was evaluated in the remaining 30% leave-out sample (184,301 individuals). Using the entire data set to predict the Phenotype Associated with an AVA <1.0 cm^2^, the area under the receiver operating characteristic curve (AUC) in the test sample was 0.986, 95% CI 0.985 to 0.987.^21^ Explainability analysis demonstrated that variables with the greatest influence on the model’s output were consistent with those typically identified in severe AS: age, transaortic gradients, left ventricular dimensions/mass, measures of diastolic function (for example, the mitral E:e’ ratio, indexed left atrial volume), and peak tricuspid regurgitation velocity. The LVEF was not a major contributor to model performance.

Among those with a predicted probability of an AVA <1.0 cm^2^ of <0.235 (F1 threshold) in the derivation sample, quintiles of hemodynamic variables (eg aortic peak velocity and mean gradient) were used to determine the probability cutoff for identifying individuals with moderate AS (a predicted probability of >0.0625 (the F2 threshold) but <0.235 [F1 threshold]). Those below this probability cutoff (ie <0.0625) were determined to be low-probability for severe AS.

Applying these probability thresholds, 4 discrete groups were identified: 1) those with an AVA <1.0 cm^2^ with a probability over the F1 threshold (ie severe AS by guidelines and AI-DSA classification); 2) those with a probability over the F1 threshold but either not-meeting guideline criteria for severe AS or inability to classify based on partial AV profiling (ie phenotype of severe AS by AI-DSA classification but non-severe AS by guidelines); 3) those with moderate AS (ie predicted probability above the F2 but below the F1 threshold); and 4) those with low-probability for severe AS (ie predicted probability below the F2 threshold).

### Statistical analysis

A deidentified data set containing all continuous measures available in BIDMC echocardiographic data was ingested into the AI-DSA model which outputted both a predicted probability of an AVA <1.0 cm^2^ and group classifications (see above). Baseline characteristics according to groups were described using means, standard deviations, numbers, and percentages as appropriate and compared across groups using analysis of variance and chi-squared statistics. Kaplan-Meier curves were generated for time to all-cause death by group membership, censoring at the end of the follow-up period (December 31, 2020), and across-group comparisons assessed using the log-rank test. Cox-proportional hazards models were built to estimate the hazard ratio (HR) and 95% CI for time-to-death by group membership, both crude and adjusted for age, sex, race, LVEF, inpatient/outpatient status, severity of mitral, tricuspid, and aortic regurgitation, ischemic heart disease, heart failure (HF), diabetes, hypertension, receipt of percutaneous coronary intervention, receipt of CABG, peak tricuspid regurgitant velocity, and the use of anticoagulant, diuretic, renin-angiotensin-neprilysn inhibitor, antiplatelet, anti-arrhythmic, and/or beta-blocker therapy. Subsequently, rates of AVRs at 1 year by group membership were computed and Kaplan-Meier curves were generated for time to AVR using cause-specific hazard techniques to account for competing risk of death (by censoring individuals who died during follow-up). As inpatient status may confound survival estimates, analyses were repeated among those only undergoing TTE as an outpatient. Among those BIDMC participants with a calculated AVA (N = 4,115), the predicted probability of AVA <1.0 cm^2^ was used to construct receiver operating characteristic curves and the sensitivity and specificity for an AVA <1.0 cm^2^ determined at the F1 and F2 thresholds, both overall and in subgroups of LVEF (<30% and <50%). Additionally, given the broad time span over which patients could be enrolled, performance was tested in those with a TTE performed after January 1, 2012. Among the subset with low-flow, low-gradient severe AS (stroke volume index <35 mL/m^2^ and AVA <1.0 cm^2^), we tested the association of AI-DSA output and mortality. As an additional sensitivity analysis, the rates of all-cause death at 5 years and AVR at 1 year were estimated based on clinical interpretation by the TTE reader at the time of the study (eg those with severe AS by AI-DSA and clinical interpretation [group A], those with severe AS phenotype by AI-DSA only but clinically non-severe AS [group B], those with severe AS by clinical interpretation only [group C], and those with non-severe AS by both AI-DSA and clinical interpretation [group D]). Cox-proportional hazards models were used to determine the median time to death and the crude HR for 5-year death by clinical interpretation status and compared across categories using log-rank tests. AS probability was used as a response variable in a separate random forest model to evaluate the association between the AI output probabilities and individual variables presented in Table 1. Analyses were conducted using JMP Pro v 15.0 (SAS Institute, Cary, NC) using a 2-tailed *P* value <0.05 to declare significance.

**Table 1:** Baseline Profile of Included Individuals According to Prediction From the Artificial Intelligence Algorithm

	All (N = 31,141)	Severe AS Groups (n = 2,012)		Non-Severe AS Groups (n = 29,129)	
		Group 1 (n = 1,549)	Group 2 (n = 463)	Group 3 (n = 979)	Group 4 (n = 28,150)
Demographic profile					
Age, years	77.5 ± 8.3	83.0 ± 8.1	83.7 ± 7.9	82.8 ± 8.0	76.9 ± 8.1
Women, %	16,230 (52.1%)	824 (53.2%)	315 (68.0%)	688 (70.3%)	14,403 (51.2%)
White, %	25,587 (82.2%)	1428 (92.2%)	407 (87.9%)	860 (87.8%)	22,892 (81.3%)
Black, %	2,810 (9.0%)	57 (3.7%)	30 (6.5%)	64 (6.5%)	2,659 (9.5%)
Other, %	2,744 (8.8%)	64 (4.1%)	26 (5.6%)	55 (5.6%)	2,599 (9.2%)
Clinical profile					
Inpatient, %	18,503 (59.4%)	1,085 (70.1%)	346 (74.7%)	732 (74.8%)	16,340 (58.1%)
Body mass index, kg/m^2^ (n = 22,598)	27.5 ± 6.1	26.5 ± 5.5	25.6 ± 5.9	25.7 ± 5.5	27.6 ± 6.1
Systolic/Diastolic blood pressure, mm Hg (n = 30,886)	131.0 ± 21.8/69.4 ± 13.4	127.0 ± 22.7/65.7 ± 13.8	128.7 ± 22.5/65.2 ± 14.2	129.2 ± 23.0/65.3 ± 13.7	131.3 ± 21.7 69.8 ± 13.3
Heart rate, bpm (n = 31,338)	73.8 ± 15.9	74.4 ± 15.4	74.0 ± 15.6	74.9 ± 16.1	73.7 ± 15.9
eGFR, L/min/1.73 m^2^ (n = 24,317)	145.7 ± 178.6	116.2 ± 139.3	127.2 ± 121.2	146.3 ± 144.3	147.6 ± 182.2
NT-proBNP, pg/ml (n = 5,334)	2,730 (809-8,119)	6,323 (1,927-15,515)	7,252 (2,708-17,059)	6,247 (2,100-14,220)	2,442 (699-7,134)
Cardiovascular comorbidity					
Type II diabetes, %	9,363 (30.0%)	525 (33.9%)	159 (34.3%)	335 (34.2%)	8,344 (29.6%)
Hypertension, %	20,075 (64.5%)	1,183 (76.4%)	351 (75.8%)	711 (72.6%)	17,830 (63.3%)
Coronary artery disease, %	15,268 (49.0%)	1,087 (70.2%)	319 (68.9%)	611 (62.4%)	13,251 (47.1%)
Heart failure, %	12,889 (41.4%)	1,020 (65.9%)	315 (68.0%)	601 (61.4%)	10,953 (38.9%)
Aortic valve profile					
Peak velocity, m/s (n = 31,132)	1.7 ± 0.7	3.8 ± 0.8	2.8 ± 0.6	2.4 ± 0.6	1.5 ± 0.4
Mean gradient, mmHg (n = 5,343)	20.1 ± 14.5	36.4 ± 16.4	20.0 ± 7.3	17.0 ± 6.4	12.0 ± 5.5
Aortic valve area, cm^2^ (n = 4,115)	1.4 ± 0.6	0.8 ± 0.2	1.3 ± 0.2	1.3 ± 0.2	1.8 ± 0.6
≥ Moderate aortic regurgitation (n = 31,141)	282 (0.9%)	40 (2.6%)	12 (2.6%)	18 (1.8%)	212 (0.8%)
Right ventricular function					
Peak TR velocity, m/s (n = 25,027)	2.8 ± 0.5	3.0 ± 0.5	3.1 ± 0.5	3.0 ± 0.5	2.7 ± 0.5
≥ Moderate TR (n = 31,141)	4,110 (13.2%)	357 (23.1%)	138 (29.8%)	261 (26.7%)	3,354 (11.9%)
Left ventricular function and dimensions					
Left atrial volume index, mL/m^2^ (n = 2,853)	30.8 ± 11.4	38.4 ± 12.6	37.1 ± 13.5	36.4 ± 13.5	30.2 ± 11.1
LV end-diastolic dimension, cm (n = 29,903)	4.5 ± 0.8	4.5 ± 0.8	4.4 ± 0.9	4.3 ± 0.9	4.5 ± 0.8
LV end-systolic dimension, cm (n = 20,237)	2.8 ± 0.8	2.9 ± 0.9	3.0 ± 1.0	2.9 ± 1.0	2.8 ± 0.7
LV ejection fraction, % (n = 31,129)	61.7 ± 16.6	58.7 ± 19.2	52.3 ± 19.4	55.7 ± 19.1	62.2 ± 16.2
Transmitral E/e’ ratio (n = 9,458)	12.3 ± 5.6	17.3 ± 8.4	16.8 ± 7.3	17.5 ± 7.4	11.8 ± 5.0
Transmitral E/A ratio (n = 11,451)	1.1 ± 0.7	1.2 ± 0.8	1.3 ± 0.8	1.3 ± 1.0	1.1 ± 0.7
Stroke volume index, mL/m^2^ (n = 10,514)	39.1 ± 11.9	37.2 ± 12.4	35.8 ± 13.5	34.9 ± 12.1	39.3 ± 11.8
≥ Moderate mitral regurgitation, % (n = 31,141)	3,391 (10.9%)	403 (26.0%)	142 (30.7%)	243 (24.8%)	2,603 (9.3%)
Clinical management					
Anticoagulant, %	4,110 (13.2%)	234 (15.1%)	69 (14.9%)	145 (14.8%)	3,662 (13.0%)
Diuretic, %	9,271 (29.8%)	518 (33.4%)	173 (37.4%)	311 (31.8%)	8,269 (29.4%)
Neurohormonal antagonists, %	10,050 (32.3%)	457 (29.5%)	144 (31.1%)	276 (28.2%)	9,173 (32.6%)
Antiplatelet, %	11,544 (37.1%)	572 (36.9%)	181 (39.1%)	347 (35.4%)	10,444 (37.1%)
Anti-arrhythmic, %	12,270 (39.4%)	619 (40.0%)	204 (44.1%)	397 (40.6%)	11,050 (39.3%)
Beta-blocker, %	12,868 (41.3%)	617 (39.8%)	204 (44.1%)	402 (41.1%)	11,645 (41.4%)
PCI, %	1,466 (4.7%)	71 (4.6%)	29 (6.3%)	65 (6.6%)	1,301 (4.6%)
CABG, %	798 (2.6%)	58 (3.7%)	17 (3.7%)	21 (2.2%)	702 (2.5%)

Values are mean ± SD or n (%). This table shows the demographic, clinical, echocardiographic profile, and treatment profile of each category of AS according to the groupings identified by the AI-DSA without access to LVOT or dimension data. The number of individuals with complete data for each variable is indicated. Group 1 indicates individuals above the F1 threshold meeting guidelines for severe AS. Group 2 (light red) indicates individuals above the F1 threshold who did not meet guideline criteria for severe AS. Group 3 indicates those below the F1 threshold in the moderate aortic valve severity category. Group 4 indicates those below the F1 threshold in the mild or less aortic valve severity category.

AS = aortic stenosis; CABG = coronary artery bypass grafting; eGFR = estimated glomerular filtration rate; LV = left ventricular; N = number of patients; NT-proBNP = N-terminal pro-brain natriuretic peptide; PCI = percutaneous coronary intervention; TR = tricuspid regurgitation.

## Results

### Cohort characteristics

Overall, 31,141 individuals were included in the analysis (mean age 77.4 ± 8.3 years) inclusive of 16,230 (52.1%) women aged 78.2 ± 8.5 years and 14,911 men aged 76.6 ± 8.0 years, the majority of whom were Caucasian (82%) and receiving a TTE while inpatient (59%). Comorbidities were frequent with diabetes present in 30%, HF in 41%, coronary artery disease (CAD) in 49%, and hypertension in 65% (Table 1). Compared to the internal validation cohort, this external validation cohort was slightly older, reflecting inclusion of Medicare beneficiaries, had a slightly greater female predominance, and had more severe AV disease but was otherwise similar across a broad range of characteristics (Supplemental Table 2).

### Results of artificial intelligence algorithm

Of those included, 1,549 (5.0%) were classified in group 1, 463 (1.5%) in group 2, 979 (3.1%) in group 3, and 28,150 (90.4%) in group 4 by the AI-DSA. With increasing AS severity, there was a progressive increase in age, proportion of inpatients, number with cardiovascular comorbidities, and worsening AV hemodynamic profiles (Table 1). In general, those determined by the AI-DSA alone to have an AVA <1.0 cm^2^ (group 2) were overall similar to those in group 1, though were more likely to be female (68.0% vs 53.2%) and Black (6.5% vs 3.7%); both *P* < 0.050. They additionally had a lower mean blood pressure (127/66 vs 131/69 mm Hg) and estimated glomerular filtration rate (116 ± 139 vs 146 ± 179 L/min/1.73 m^2^), and higher N-terminal pro-brain natriuretic peptide values (median 6,323 [IQR: 1,927-15,515] vs 2,730 [IQR: 609-8,119]); all *P* < 0.001. Individuals in group 2 also had a higher AVA (1.3 ± 0.2 vs 0.8 ± 0.2 cm^2^), a lower aortic peak velocity (2.8 ± 0.6 vs 3.8 ± 0.8 m/s), and a lower mean gradient (20.0 ± 7.3 vs 36.4 ± 16.4 mm Hg) than those in group 1; *P* < 0.001 for all. They additionally had worse LV systolic function (LVEF 52.3 ± 19.4% vs 58.7 ± 19.2%; *P* < 0.001) and more moderate or greater mitral regurgitation (30.7% vs 26.0%; *P* < 0.001). Aortic valve parameters indicated an AVA in the moderate range (1.3 ± 0.2 cm^2^) in group 3 and in the mild range (1.8 ± 0.6 cm^2^) in group 4.

### Artificial intelligence algorithm performance

**Figure 2:**
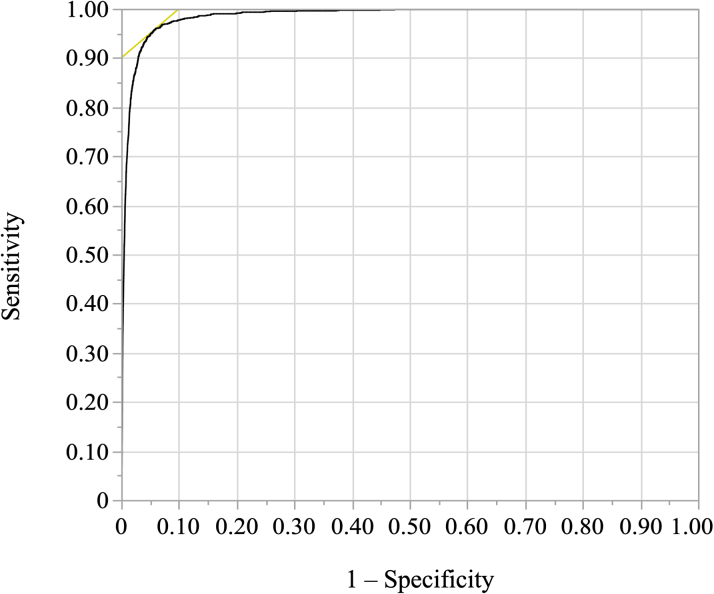
**Receiver Operating Characteristic Curve for the Detection of Severe Aortic Stenosis** Shown is the receiver operating characteristic curve for the detection of the severe aortic stenosis phenotype (Defined by echocardiographic features that associate with an AVA<1 cm^2^) using the outputted probability produced by the AI-DSA (Using the F1 threshold of a probability >0.235). The area under the curve was 0.986 indicating excellent discrimination ability. AI-DSA = artificial intelligence decision support algorithm; other abbreviation as in Figure 1.

A total of 4,115/31,141 (13.2%) individuals had AVA measurements. As it is laboratory policy at BIDMC to report AVA only when the aortic peak velocity >2 m/s, this number likely accounts for all those with an AVA <1.0 cm^2^. Of these, 1,422 (34.6%) had an AVA <1.0 cm^2^. The AUC for detection of an AVA <1.0 cm^2^ was 0.986 (Figure 2), similar to that of the internal validation cohort.^21^ Using the F1 threshold of >0.235 to define severe AS, the sensitivity, specificity, positive predictive value, and negative predictive value (NPV) were 82.2%, 98.1%, 67.4%, and 99.2%, respectively. Using the F2 threshold of >0.0625, the sensitivity, specificity, positive predictive value, and NPV were 95.2%, 94.8%, 46.4%, and 99.8%, respectively. Using the F1 threshold, post hoc analysis among the subset of cases with a LVEF <50% (N = 5,966) or LVEF <30% (N = 1,611), the AI-DSA continued to perform well to identify an AVA <1.0 cm^2^ with an overall AUC of 0.984 (sensitivity 87.2%, specificity 97.1%) and 0.981 (sensitivity 89.4%, specificity 95.6%), respectively, though slightly lower than the internal validation cohort (AUC = 0.986 for LVEF <50% and AUC = 0.981 for LVEF <30%). Using the F1 threshold, among those who received a TTE in after 2012 (N = 17,372), AI-DSA performance continued to perform well to identify an AVA <1.0 cm^2^ with an AUC of 0.991 (sensitivity 95.9%, specificity 95.9%). In the subset with low-flow, low-gradient severe AS (N = 521), adjusting for age, sex, and race, the AI-DSA output continued to be associated with mortality (HR: 1.49; 95% CI: 1.17-1.89; *P* = 0.001).

### Aortic valve replacement

Overall, 640 (2.1%) individuals underwent AVR at a median 342 (IQR: 26-1,318) days after TTE. A total of 215 (0.7%) underwent TAVR at a median 1,189 (IQR: 691-2,478) days and 432 (1.4%) underwent surgical aortic valve replacement at a median 75 (IQR: 7-623) days after TTE. Individuals in groups 1 and 2 who underwent AVR were younger, predominately male, had higher AV gradients, more frequently had CAD and CABG, and less frequently had HF or mitral and tricuspid regurgitation (all *P* < 0.050) (Supplemental Table 3). A total of 340 (21.9%) of group 1, 20 (4.3%) of group 2, 49 (5.0%) of group 3, and 231 (1.5%) of group 4 underwent AVR (Table 2). Of TTEs performed after 2011 (N = 17,372), 201 (25.2%) of those in group 1, <11 in group 2, 32 (6.9%) in group 3, and 123 (0.8%) in group 4 underwent AVR. A total of 325 (50.8%) of AVRs occurred within 1 year of TTE (Supplemental Table 4). Among those who underwent any AVR, 281 (43.9%) died at a median 5.0 (IQR: 2.3-8.4) years after TTE compared to 18,165 (59.6%) of those who did not undergo AVR (HR: 0.55; 95% CI: 0.49-0.62; *P* < 0.001) (Supplemental Figure 1).

**Table 2:** Rates of Timing of Aortic Valve Replacement After Index Echocardiogram According to Predicted Groups

	Severe AS Groups (n = 2,012)		Non-Severe AS Groups (n = 29,129)	
	Group 1 (n = 1,549)	Group 2 (n = 463)	Group 3 (n = 979)	Group 4 (n = 28,150)
All aortic valve replacements				
Number of cases, %	340 (21.9%)	20 (4.3%)	49 (5.0%)	231 (1.5%)
Median (IQR) time to procedure, days	69 (7-355)	292 (9-1,322)	797 (271-1,450)	1,504 (598-2,620)
Surgical aortic valve replacement				
Number of cases, %	261 (16.8%)	12 (2.6%)	31 (3.2%)	128 (0.5%)
Median (IQR) time to procedure, days	33 (5-157)	11 (5-196)	585 (92-1,055)	708 (73-1,729)
Transcatheter aortic valve replacement				
Number of cases, %	81 (5.2%)	< 11	18 (1.8%)	108 (0.4%)
Median (IQR) time to procedure, days	512 (174-894)	1,387 (953-2,593)	1,202 (797-2,434)	2238 (1,499-3,675)

This table shows the number and timing of aortic valve replacement (AVR) by according to predicted AI-DSA group. Overall AVRs are presented as well as stratified by surgical or transcatheter aortic valve receipt. Group 1 indicates individuals above the F1 threshold meeting guidelines for severe AS. Group 2 indicates individuals above the F1 threshold who did not meet guideline criteria for severe AS. Group 3 indicates those below the F1 threshold in the moderate aortic valve severity category. Group 4 indicates those below the F1 threshold in the mild or less aortic valve severity category.

AI-DSA = artificial intelligence decision support algorithm; other abbreviation as in Table 1. Cell numbers under 11 are suppressed by Medicare data use policy.

### Mortality results

**Figure 3:**
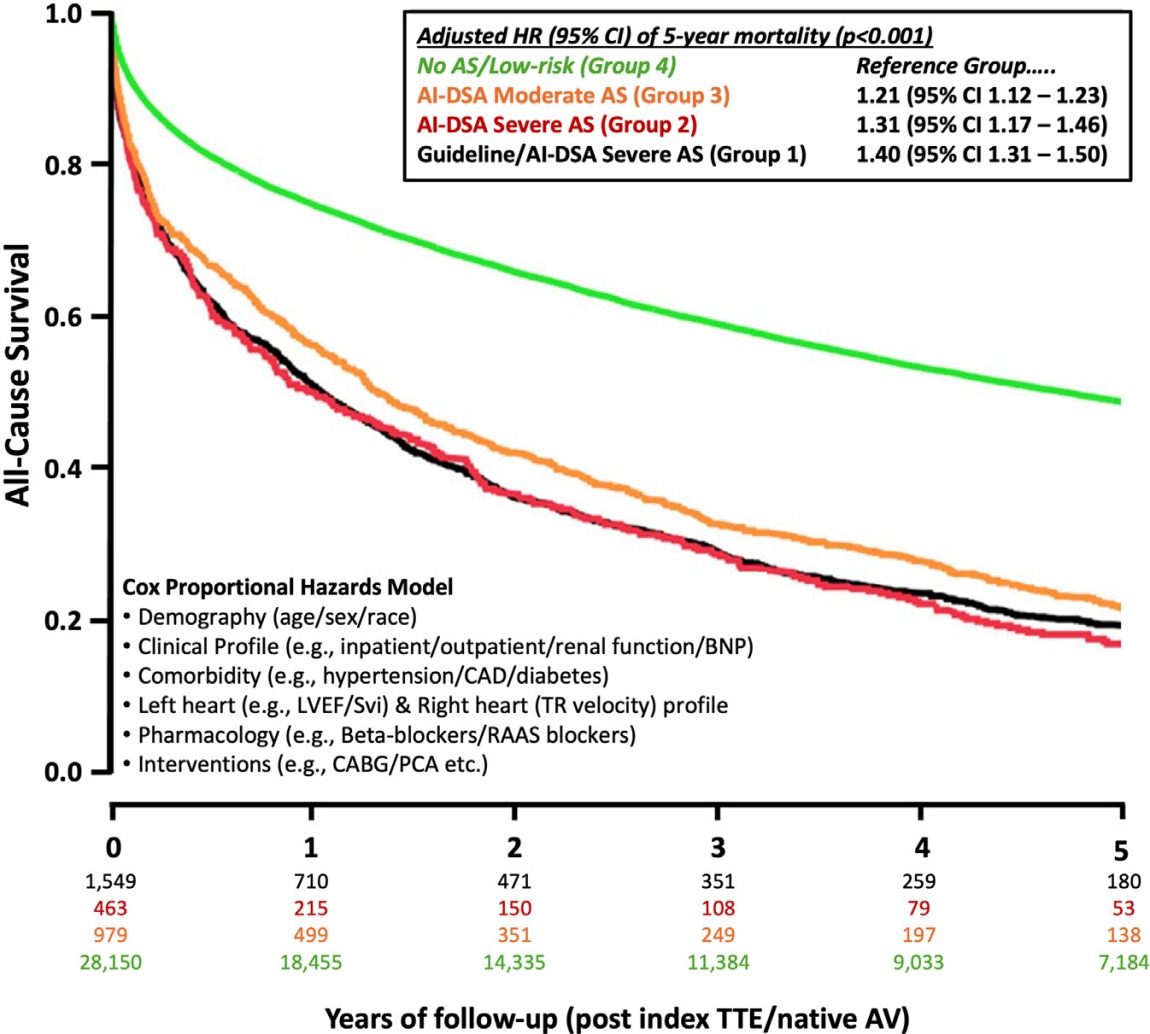
**Kaplan-Meier Curve Demonstrating Time to All-Cause Mortality by Artificial Intelligence Algorithm Grouping** Displayed is a kaplan-meier curve demonstrating time from the index echocardiogram to all-cause mortality by AI-DSA group. Numbers in the risk set at one-year time points are provided below. Group 1 (Black Line) indicates individuals above the F1 threshold meeting guidelines for severe AS. Group 2 (Red Line) indicates individuals above the F1 threshold who did not meet guideline criteria for severe AS. Group 3 (Orange Line) indicates those below the F1 threshold in the moderate aortic valve severity category. Group 4 (Green Line) indicates those below the F1 threshold in the mild or less aortic valve severity category. The log-rank p-value for comparison across groups was <0.001. CABG = coronary artery bypass grafting; CAD = coronary artery disease; PCI = percutaneous coronary intervention; TR = tricuspid regurgitation; TTE = transthoracic echocardiogram; other abbreviations as in Figures 1 and 2.

Over a median 8.1 (IQR: 5.0-12.1) years of follow-up, 18,446 (59.2%) individuals died at a median 5.5 (IQR: 1.0-12.8) years after TTE. Of these deaths, 14,482 (78.5%) occurred within 5 years of the index echocardiogram. Five-year mortality was highest in group 2 (77.1%) followed by group 1 (73.9%), group 3 (71.2%), and group 4 (43.6%) (Figure 3, log-rank *P* < 0.001 for overall between group comparison, *P* = 0.008 for comparison of group 1-2). After multivariable adjustment for age, sex, race, LVEF, presence of CAD, HF, revascularization for CAD, and 14 other echocardiographic and treatment variables, individuals in groups 1 to 3 were significantly more likely to die at 5 years than those in group 4 (group 1, adjusted hazard ratios [aHR]: 1.40; 95% CI: 1.31-1.50; group 2, aHR: 1.31; 95% CI: 1.17-1.46; group 3, aHR: 1.21; 95% CI: 1.12-1.32; all *P* < 0.001).

Among 12,638 (40.2%) outpatients, there were 5,035 (39.8%) deaths at a median 10.5 (IQR: 4.1-16.8) years after TTE. Five-year mortality was highest in group 1 (56.5%) followed by group 2 (53.0%), group 3 (46.6%), and group 4 (25.4%) (Supplemental Figure 2) (log-rank *P* < 0.001). After similar multivariate adjustment, individuals in groups 1 to 3 continued to be at higher risk compared to group 4 with adjusted HRs ranging 1.32 to 1.67.

### Comparison with clinical evaluation

A total of 978 (3.1%) individuals had the severe AS phenotype by both AI-DSA and clinical interpretation (group A), 1,034 (3.3%) by AI-DSA only (group B), <11 by clinical interpretation only (group C), and 29,124 (93.5%) had non-severe AS by both AI-DSA and clinical interpretation (group D). While individuals in group A and B were similar across many clinical and TTE features, group B had AV parameters similar to those with moderate AS (Supplemental Table 5). A total of 742 (75.9%) individuals in group A, 760 (73.5%) in group B, <11 in group C, and 12,978 (44.6%) in group D died at 5 years after TTE (Supplemental Table 6). Compared to group D, both groups A (HR: 2.48; 95% CI: 2.30-2.67) and B (HR: 2.21; 95% CI: 2.06-2.28) had elevated mortality risk (both *P* < 0.001). A total of 199 (20.4%) in group A, 68 (6.6%) in group B, 0 (0.0%) in group C, and 58 (0.2%) in group D received an AVR at 1 year after TTE. Random forest models demonstrated that the probability output was associated with a number of clinical and echocardiographic variables typically relevant in the clinical assessment of patients with severe AS. After exclusion of AV parameters, the top 10 variables from the random forest plot were age, peak tricuspid regurgitant velocity, mitral regurgitation severity, blood pressure, LVEF, stroke volume index, estimated glomerular filtration rate, left ventricular diastolic diameter, body mass index, and transmitral E/e’ ratio (Supplemental Table 7).

## Discussion

In a large cohort of North American individuals >65 years undergoing TTE, a tested and validated AI-DSA using only echocardiographic report variables showed high performance in detecting the phenotype associated with an AVA <1 cm^2^, despite the exclusion of LVOT measurement. The AI-DSA enhanced clinical evaluation of severe AS by highlighting all patients that may have been reported on echo, along with an additional and equal proportion of patients with the severe AS phenotype but predominantly did not meet current clinical guidelines. Such individuals had a similar mortality risk to those clinically determined to have severe AS but were substantially less likely to receive AVR. Overall rates of AVR remained low, even in those with a reported AVA <1 cm^2^ (21.9%), despite those receiving AVR showing improved mortality. These results suggest a possible utility for this AI-DSA alongside standard echo reporting to enhance the detection of individuals with a severe AS phenotype, at risk for adverse outcomes (Central Illustration).

**Central Illustration:**
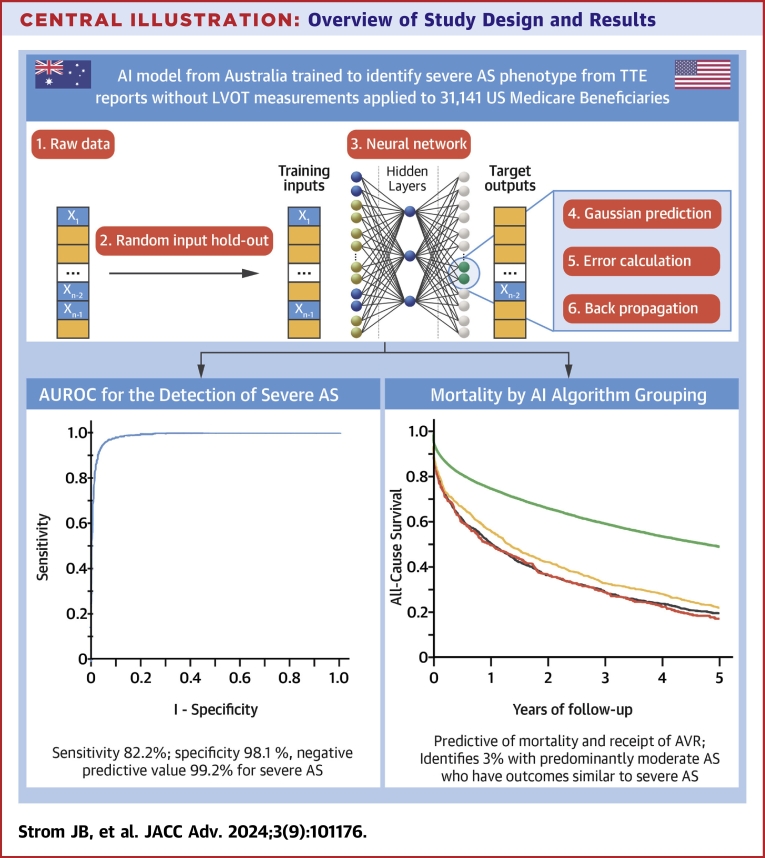
**Overview of Study Design and Results** This diagram demonstrates the derivation and external validation of an Artificial Intelligence (AI) model that uses TTE routine TTE reports to identify the severe aortic stenosis (AS) phenotype, not requiring LVOT measurements. Model results were not only predictive of mortality and receipt of AVR but identified an additional 3% of individuals with predominantly moderate AS who had outcomes similar to that of severe AS and low rates of AVR. ROC = receiver operating characteristic; other abbreviations as in Figures 1 and 3.

Numerous studies have shown that severe AS remains undertreated with only ∼30% of those with symptomatic severe AS eventually undergoing AVR,^1^^,^^7^, ^8^, ^9^, ^10^ despite lifesaving benefits. The most common reason for non-referral to AVR is that presence of symptoms is not attributed to the underlying AS ^7^ despite recent data that bring this into question.^29^ Furthermore, inaccurate echocardiographic measurement of AS severity, in part due to reliance on error-prone LVOT measurements to determine AVA may limit the number of individuals correctly classified as having severe AS.^30^ As the population ages and treatments for AS become increasingly widespread,^31^, ^32^, ^33^ a growing number of patients will require accurate and reproducible echocardiography-guided decision-making.^34^ In this setting, decision support tools are needed to assist clinicians in the challenging but important task of determining one’s AS severity.^35^ Moreover, even among referred patients, wait times for AVR can range from 12 to 20 weeks ^9^^,^^11^^,^^36^^,^^37^ with a relative 2% increase in 1-year mortality per week delay after initial referral,^37^ suggesting the need to identify individuals at high risk for prompt referral/treatment when indicated.

In this study, an AI-DSA trained on data from over 1 million echocardiogram reports across 23 sites in Australia showed a high sensitivity (82.2%) and specificity (98.1%) in identifying individuals with an AVA <1.0 cm^2^ when blindly applied to TTE data from 31,141 individuals at BIDMC, despite imbalances between groups. Furthermore, the high NPV (99.2%) suggests that this algorithm could effectively be used to rule out severe AS, using minimal input information. This performance continued to be robust in those with depressed LV function and more recently performed TTEs. This study mirrors findings by Sengupta et al. in which TTE report data alone were used to identify the AS phenotype and similarly showed to be prognostic of adverse outcomes.^19^ However, by contrast to this prior study, the current algorithm does not require information on indexed AVA, stroke volume index, or aortic mean gradient to generate an output, and thus may be useful in cases that these data are not available.^19^

While the AI-DSA identified individuals meeting current guideline-criteria for severe AS (group 1), it additionally identified 463 individuals (1.5%, group 2) with a similar predicted probability of severe AS but not meeting guideline criteria for severity. These individuals had a similar clinical profile as those in group 1, but tended to have lower AV gradients. Of note, despite similar if not higher 5-year mortality than those in group 1, this group received AVRs at a significantly lower rate (4.3% vs 21.9%). In general, though increased over time, rates of AVR, even in patients meeting guideline criteria for severe AS remained low, consistent with prior published data.^1^^,^^7^, ^8^, ^9^, ^10^ As use of claims to identify AVR is both valid and captures AVRs performed at any U.S. site,^26^ this number likely represents true estimates for treatment. Those who underwent AVR were younger and less frequently had HF or other significant valvular heart disease, suggesting underlying selection bias in the choice of referring for AVR. Moreover, death rates were substantially lower in this population, which may represent treatment selection as well as a true treatment effect.

Validating against routine clinical evaluation, the AI-DSA identified nearly all of those with severe AS by clinical interpretation with <11 patients not identified. Furthermore, the AI-DSA identified an additional 1,034 (3.3%) individuals as high probability of having the severe AS phenotype (group B) with predominantly moderate AS by clinical guidelines. They also had similar mortality risk as those identified as having severe AS by clinical interpretation though received AVRs less frequently (6.6% vs 20.3%). While it is not possible to conclude that these group B individuals would have benefitted from AVR, future prospective testing to assess the utility of following an AI-DSA for identifying severe AS vs a routine clinical interpretation strategy is underway and will help identify how use of this software impacts referrals, testing, clinical management, and outcomes. To improve clinical reasoning, software under development for clinical use will present users with a screen listing all clinical variables used for prediction. While these study results remain promising, prospective testing in a clinical environment is necessary so that these algorithms improve upon existing practice.

### Study Limitations

While large and representative of TTEs performed in routine practice, the current study has several limitations to consider. First, as a retrospective study, causality cannot be inferred with the current methods and thus prospective testing is required and ongoing to assess if an AI-DSA driven strategy may improve patient outcomes. Second, due to the large number of participants, it was not feasible to review raw images to verify measurements. However, these measurements are reflective of actual values given in clinical practice and in large numbers; the values may approximate population means.^38^ Third, covariates and AVR rates were determined via claims-ascertainment and thus, while validated for use in these circumstances,^25^^,^^26^ misclassification of covariate status is possible. This was considered outside the scope of the current study. Fourth, as the study included Medicare beneficiaries ≥65 years, the performance of this AI-DSA in the <65 population cannot be assessed in the current study. Nevertheless, as the majority of cases of AS occur in individuals ≥65 years old, it is likely that results generalize to most individuals with AS. Furthermore, as the derivation and initial testing of the AI-DSA was performed across >30 centers in Australia among those with clinically indicated echocardiography of all ages, the algorithm is likely to generalize to a broader population. Fifth, as routinely collected TTE variables are similar in their definitions/quantification, domain adaptation was not applied, and it remains unknown if this would improve model performance in the external data set. Sixth, while the inclusion of mortality outcomes was intended to support the notion that the AI-DSA classification is clinically meaningful, cause of death information is not available, and it is not possible to attribute these deaths to AS. Seventh, it is not possible to conclude that those with non-severe AS but a similar phenotype to those with severe AS would derive benefit from AVR. Eighth, while AVA measurements were not necessary for the AI-DSA model output, the ability to clinically identify individuals with an AVA <1.0 cm^2^ was influenced by available data on AVA measurements. As significant AS is uncommonly seen with an aortic peak velocity <2 m/s, it is unlikely that this selection introduces significant bias into the cohort but prospective testing is necessary to fully evaluate model performance in a clinical setting.

## Conclusions

In this large cohort of patients >65 years undergoing TTE, a previously derived AI-DSA using only echocardiographic measurement report variables showed high performance in detection of individuals with the phenotype of an AVA <1 cm^2^, despite the exclusion of the LVOT measurement. These results suggest a possible utility for this AI-DSA to be used in parallel to standard echocardiography reporting to enhance detection of individuals with the phenotype of severe AS, at risk for adverse outcomes.

Perspectives **COMPETENCY IN MEDICAL KNOWLEDGE:** Up to 67% of patients with severe AS do not undergo AVR. Moreover, it is challenging to identify moderate AS patients with a similar mortality risk to those with severe AS.

**TRANSLATIONAL OUTLOOK 1:** An AI-DSA applied to routine echocardiogram reports from U.S. Medicare beneficiaries had a sensitivity, specificity, and NPV to detect the phenotype associated with severe AS (AVA <1 cm^2^) of 82.2%, 98.1%, and 99.2%, respectively, without relying on LVOT measurements. The AI-DSA additionally identified individuals with predominantly moderate AS with a similar phenotype and outcome of those with severe AS.

**TRANSLATIONAL OUTLOOK 2:** These results overall suggest possible utility for this AI-DSA to enhance detection of individuals with severe AS at risk for adverse outcomes.

## Funding support and author disclosures

This work was supported by Echo IQ Pty Ltd. Dr Stewart is supported by the 10.13039/501100000925National Health and Medical Research Council of Australia (GNT1135894). Dr Strom is supported by the 10.13039/100000002National Institutes of Health (1K23HL144907, R01AG063937), 10.13039/100006520Edwards Lifesciences, Ultromics, HeartSciences, Anumana, and EchoIQ. Unrelated to this work, Dr Strom has served on the Scientific Advisory Board for Edwards Lifesciences and EchoIQ and has received consulting fees from Bracco Diagnostics, General Electric Healthcare, and Lantheus Medical Imaging. Prof Strange has received consulting fees from Edwards, Medtronic, and Echo IQ and has received speaker fees from Edwards, Medtronic, Abbott, and Echo IQ. Prof Playford has received consulting fees from Edwards, Medtronic, and Echo IQ. Prof Stewart has received consulting fees from Echo IQ.
