# Supplementary material for: An Artificial Intelligence Algorithm for Detection of Severe Aortic Stenosis: A Clinical Cohort Study
Source: JACC Adv. 2024 Sep 25;3(9):101176. doi: 10.1016/j.jacadv.2024.101176 (PMC11450902; doi:10.1016/j.jacadv.2024.101176)
Supplement: Supplemental material [file mmc1.docx]

Supplementary Appendix

**Supplementary Table 1:** Echocardiographic Variables Used as Model Inputs for AI-DSA Derivation

| **Variable name** | **Data type** | **Unit Name** | **Label** | **Long description** |
| --- | --- | --- | --- | --- |
| StudyID | Numeric | Numeric | Study ID | Unique record identifier |
| PatientID | Numeric | Numeric | Patient ID | Unique patient identifier, repeats for patients with multiple echos |
| Age_At_Echo | Numeric | Numeric | Age at Echo | Age at time of echo |
| Genderfinal | Numeric | Numeric | Patient Gender | Gender |
| Aorta_at_Sinotubular_Diameter | Numeric | cm | Aorta at Sinotubular Diameter | Aorta at Sinotubular Diameter |
| Aorta_at_Sinuses_Diameter | Numeric | cm | Aorta at Sinuses Diameter | Aorta at Sinuses Diameter |
| Aortic_Arch_Diameter | Numeric | cm | Aortic Arch Diameter | Aortic Arch Diameter |
| Aortic_Root_Diameter_MM | Numeric | cm | Aortic Root Diameter MM | Aortic Root Diameter M-Mode |
| Aortic_Root_Diameter | Numeric | cm | Aortic Root Diameter | Aortic Root Diameter |
| AR_Pressure_Half_Time | Numeric | ms | AR Pressure Half Time | Aortic Regurgitation Pressure Half Time |
| AR_Slope | Numeric | cm/s² | AR Slope | Aortic Regurgitation Slope |
| Ascending_Aorta_Diameter | Numeric | cm | Ascending Aorta Diameter | Ascending Aorta Diameter |
| AV_Diameter | Numeric | cm | AV Diameter | Aortic Valve Diameter |
| AV_Mean_Gradient | Numeric | mmHg | AV Mean Gradient | Aortic Valve Mean Gradient |
| AV_Mean_Velocity | Numeric | cm/s | AV Mean Velocity | Aortic Valve Mean Velocity |
| AV_Peak_Gradient | Numeric | mmHg | AV Peak Gradient | Aortic Valve Peak Gradient |
| AV_Peak_Velocity | Numeric | cm/s | AV Peak Velocity | Aortic Valve Peak Velocity |
| AV_Stroke_Volume | Numeric | cm³ | AV Stroke Volume | Aortic Valve Stroke Volume |
| AV_Velocity_Ejection_Time | Numeric | ms | AV Velocity Ejection Time | Aortic Valve Velocity Ejection Time |
| AV_Velocity_Time_Integral | Numeric | cm | AV Velocity Time Integral | Aortic Valve Velocity Time Integral |
| AVmeangradienttext | Numeric | mmHg | AV Mean Gradient Text | Aortic valve mean gradient reported in text of report |
| AVmeanvelocitytext | Numeric | cm/s | AV Mean Velocity Text | Aortic valve mean velocity reported in text of report |
| AVpeakgradienttext | Numeric |  | AV Peak Gradient Text | Aortic valve peak gradient reported in text of report |
| AVpeakvelocitytext | Numeric | cm/s | AV Peak Velocity Text | Aortic valve peak velocity reported in text of report |
| Body_Height | Numeric | cm | Body Height | Body Height |
| Body_Mass_Index_calculated | Numeric | kg/m² | Body Mass Index (kg/m²) | Body Mass Index calculated |
| Body_Surface_Area | Numeric | m² | Body Surface Area formula SQRT (Height x Wt/3600) | Body Surface Area |
| Body_Weight | Numeric | kg | Body Weight | Body Weight |
| Heart_Rate | Numeric | bpm | Heart Rate | Heart Rate |
| Isovolumic_Relaxation_Time | Numeric | ms | Isovolumic Relaxation Time | Isovolumic Relaxation Time |
| IVC_Diameter_Expiration | Numeric | cm | IVC Diameter Expiration | IVC Diameter Expiration |
| IVC_Diameter_Inspiration | Numeric | cm | IVC Diameter Inspiration | IVC Diameter Inspiration |
| IVS_Diastolic_Thickness_MM | Numeric | cm | IVS Diastolic Thickness MM | IVS Diastolic Thickness M-Mode |
| IVS_Diastolic_Thickness | Numeric | cm | IVS Diastolic Thickness | IVS Diastolic Thickness |
| IVS_Systolic_Thickness_MM | Numeric | cm | IVS Systolic Thickness MM | IVS Systolic Thickness M-Mode |
| IVS_Systolic_Thickness | Numeric | cm | IVS Systolic Thickness | IVS Systolic Thickness |
| IVS_to_PW_Ratio | Numeric | unitless | IVS to PW Ratio | IVS to PW Ratio |
| LA_Ao_Ratio_MM | Numeric | unitless | LA Ao Ratio MM | LA Ao Ratio M-Mode |
| LA_Ao_Ratio | Numeric | cm/s² | LA Ao Ratio | LA Ao Ratio |
| LA_Area_2C_View | Numeric | cm² | LA Area 2C View | LA Area 2C View |
| LA_Area | Numeric | cm² | LA Area | LA Area 4C View |
| LA_Length_2C | Numeric | cm | LA Length 2C | LA Length 2C View |
| LA_Length_4C | Numeric | cm | LA Length 4C | LA Length 4C View |
| LA_Systolic_Diameter_LX | Numeric | cm | LA Systolic Diameter LX | LA Systolic Diameter Parasternal Long Axis |
| LA_Systolic_Diameter_MM | Numeric | cm | LA Systolic Diameter MM | LA Systolic Diameter M-Mode |
| LA_Systolic_Diameter_Transverse | Numeric | cm | LA Systolic Diameter Transverse | LA Systolic Diameter Transverse |
| LA_Volume_Index | Numeric | cm³/m² | LA Volume Index | LA Volume Index |
| LA_Volume | Numeric | cm³ | LA Volume | LA Volume |
| LAVItext | Numeric | ml/m² | LAVI Text | LA Volume Index extracted from text reporting |
| LV_Diast_Vol_2D_Teich | Numeric | cm³ | LV diastolic volume 2D Teichholz | LV Diastolic Volume 2D Teichholz method |
| LV_Diast_Vol_MM_Teich | Numeric | cm³ | LV diastolic volume M mode Teichholz | LV Diastolic Volume M-Mode Teichholz method |
| LV_Diastolic_Area_2C | Numeric | cm² | LV Diastolic Area 2C | LV Diastolic Area 2C - MOD |
| LV_Diastolic_Area_4C | Numeric | cm² | LV Diastolic Area 4C | LV Diastolic Area 4C - MOD |
| LV_Diastolic_Area_PSAX | Numeric | cm² | LV Diastolic Area PSAX | LV Diastolic Area PSAX for LV mass assessment |
| LV_Diastolic_Diameter_4C | Numeric | cm | LV Diastolic Diameter 4C | LV Diastolic Diameter 4C |
| LV_Diastolic_Diameter_MM | Numeric | cm | LV Diastolic Diameter MM | LV Diastolic Diameter M-Mode |
| LV_Diastolic_Diameter_PLAX | Numeric | cm | LV Diastolic Diameter PLAX | LV Diastolic Diameter PLAX |
| LV_Diastolic_Length_2C | Numeric | cm | LV Diastolic Length 2C | LV Diastolic Length 2C |
| LV_Diastolic_Length_4C | Numeric | cm | LV Diastolic Length 4C | LV Diastolic Length 4C |
| LV_Diastolic_Volume_2C_AL | Numeric | cm³ | LV Diastolic Volume 2C AL | LV Diastolic Volume 2C Area Length method |
| LV_Diastolic_Volume_4C_AL | Numeric | cm³ | LV Diastolic Volume 4C AL | LV Diastolic Volume 4C Area Length method |
| LV_Diastolic_Volume_MOD_2C | Numeric | ml | LV Diastolic Volume MOD 2C | LV Diastolic Volume MOD 2C method |
| LV_Diastolic_Volume_MOD_4C | Numeric | ml | LV Diastolic Volume MOD 4C | LV Diastolic Volume MOD 4C method |
| LV_Diastolic_Volume_MOD_BP | Numeric | cm³ | LV Diastolic Volume MOD BP | LV Diastolic Volume MOD BP method |
| LV_Diastolic_Volume_SIM | Numeric | cm³ | LV Diastolic Volume SIM | LV Diastolic Volume SIM method |
| LV_Ejection_Fraction_MMode_Teich | Numeric | % | LV Ejection Fraction M mode Teichholz | LV Ejection Fraction M-Mode Teichholz Method |
| LV_Ejection_Fraction_2D_Teich | Numeric | unitless | LV Ejection Fraction 2D Teichholz | LV Ejection Fraction 2D Teichholz Method |
| LV_Ejection_Fraction_MOD_2C | Numeric | % | LV Ejection Fraction MOD 2-chamber | LV Ejection Fraction 2C MOD Method |
| LV_Ejection_Fraction_MOD_4C | Numeric | % | LV Ejection Fraction MOD 4-chamber | LV Ejection Fraction 4C MOD Method |
| LV_Ejection_Fraction_MOD_BP | Numeric | % | LV Ejection Fraction MOD Apical Biplane | LV Ejection Fraction Biplane MOD Method |
| LV_EFtext | Numeric | % | LV Ejection Fraction From Text Extraction | Ejection Fraction reported in text of report |
| LV_Ejection_Fraction_Hierarchy | Numeric | unitless | LV Ejection Fraction | Ejection Fraction - Based on hierarchy (EF text, biplane, 4C, 2C, Teicholz, M-mode) |
| LV_Epi_Diastolic_Area_PSAX | Numeric | cm² | LV Epi Diastolic Area PSAX | LV Epicardial Diastolic Area PSAX for LV mass assessment |
| LV_Endocardial_Area_PSAX | Numeric |  | LV Endocardial Area PSAX |  |
| LV_Fractional_Shortening_MM | Numeric | unitless | LV Fractional Shortening MM | LV Fractional Shortening M-Mode |
| LV_Fractional_Shortening_PLAX | Numeric | unitless | LV Fractional Shortening PLAX | LV Fractional Shortening PLAX |
| LV_Lateral_E_Prime_Velocity | Numeric |  | MV Lateral E Prime Velocity |  |
| LV_Mass_2D_ASE | Numeric |  | LV Mass Basal 2D ASE method |  |
| LV_Mass_2D_ASE_Index | Numeric |  | LV Mass Basal 2D ASE method Indexed |  |
| LVH_2D_ASE | Numeric |  | LVH Basal 2D (corrected for gender) |  |
| LV_Mass_AL_Index | Numeric | g/m² | LV Mass AL Index | LV Mass Index Area Length Method |
| LV_Mass_AL | Numeric | g | LV Mass AL | LV Mass Area Length Method |
| LV_Mass_MM | Numeric | g | LV Mass MM | LV Mass M-Mode |
| LV_Mass_Index_MM | Numeric |  | LV Mass Index MM | LV Index lv_mass_index |
| LV_Mitral_E_to_E_Prime_Ratio_Hierarchy | Numeric | unitless | Mitral E to E Prime Ratio Combined | Medial Mitral Annular Tissue Doppler E to E Prime Ratio - Based on hierarchy (E:E' ratio text, Mitral E to MV E' Ratio) |
| LV_Mitral_E_to_LV_E_Prime_Lateral_Ratio | Numeric | unitless | Mitral E to LV Lateral E Prime Ratio | Lateral Mitral Annular Tissue Doppler E to E Prime Ratio |
| LV_Mitral_E_to_MV_E_Prime_Septal_Ratio | Numeric | unitless | Mitral E to MV Septal E Prime Ratio | Medial Mitral Annular Tissue Doppler E to E Prime Ratio |
| LV_Relative_Wall_Thickness_MM | Numeric | unitless | LV Relative Wall Thickness MM | LV Relative Wall Thickness M-Mode |
| LV_Relative_Wall_Thickness | Numeric | cm | LV Relative Wall Thickness | LV Relative Wall Thickness |
| LV_Septal_E_Prime_Velocity | Numeric |  | LV Septal E Prime Velocity |  |
| LV_Stroke_Volume_2D_Teich | Numeric | cm³ | LV Stroke Volume 2D Teich | LV Stroke Volume 2D Teichholz Method |
| LV_Stroke_Volume_4C_AL | Numeric | cm³ | LV Stroke Volume 4C AL | LV Stroke Volume 4C Area Length Method |
| LV_Stroke_Volume_MM_Teich | Numeric | cm³ | LV Stroke Volume MM Teich | LV Stroke Volume M-Mode Teichholz Method |
| LV_Stroke_Volume_MOD_2C | Numeric | cm³ | LV Stroke Volume MOD 2C | LV Stroke Volume MOD 2C Method |
| LV_Stroke_Volume_MOD_4C | Numeric | cm³ | LV Stroke Volume MOD 4C | LV Stroke Volume MOD 4C Method |
| LV_Stroke_Volume_MOD_BP | Numeric | cm³ | LV Stroke Volume MOD BP | LV Stroke Volume MOD BP Method |
| LV_Stroke_Volume_SIM | Numeric | cm³ | LV Stroke Volume SIM | LV Stroke Volume SIMPSON'S Method |
| LV_Syst_Vol_2D_Teich | Numeric | cm³ | LV systolic volume 2D Teichholz | LV Systolic Volume 2D Teichholz Method |
| LV_Syst_Vol_MM_Teich | Numeric |  | LV systolic volume M mode Teichholz |  |
| LV_Systolic_Area_2C | Numeric | cm² | LV Systolic Area 2C | LV Systolic Area 2C |
| LV_Systolic_Area_4C | Numeric | cm² | LV Systolic Area 4C | LV Systolic Area 4C |
| LV_Systolic_Diameter_4C | Numeric | cm | LV Systolic Diameter 4C | LV Systolic Diameter 4C |
| LV_Systolic_Diameter_MM | Numeric | cm | LV Systolic Diameter MM | LV Systolic Diameter M-Mode |
| LV_Systolic_Diameter_PLAX | Numeric | cm | LV Systolic Diameter PLAX | LV Systolic Diameter Base Parasternal Long Axis View |
| LV_Systolic_Length_2C | Numeric | cm | LV Systolic Length 2C | LV Systolic Length 2C |
| LV_Systolic_Length_4C | Numeric | cm | LV Systolic Length 4C | LV Systolic Length 4C |
| LV_Systolic_Volume_2C_AL | Numeric | cm³ | LV Systolic Volume 2C AL | LV Systolic Volume 2C Area Length Method |
| LV_Systolic_Volume_4C_AL | Numeric | cm³ | LV Systolic Volume 4C AL | LV Systolic Volume 4C Area Length Method |
| LV_Systolic_Volume_MOD_2C | Numeric | ml | LV Systolic Volume MOD 2C | LV Systolic Volume MOD 2C Method |
| LV_Systolic_Volume_MOD_4C | Numeric | ml | LV Systolic Volume MOD 4C | LV Systolic Volume MOD 4C Method |
| LV_Systolic_Volume_MOD_BP | Numeric | cm³ | LV Systolic Volume MOD BP | LV Systolic Volume MOD BP Method |
| LV_Systolic_Volume_SIM | Numeric | cm³ | LV Systolic Volume SIM | LV Systolic Volume SIMPSON'S Method |
| LVPW_Diastolic_Thickness_MM | Numeric | cm | LVPW Diastolic Thickness MM | LVPW Diastolic Thickness M-Mode |
| LVPW_Diastolic_Thickness | Numeric | cm | LVPW Diastolic Thickness | LVPW Diastolic Thickness |
| Mitral_A_Duration | Numeric | ms | Mitral A Duration | Mitral A Duration |
| Mitral_A_Point_Velocity | Numeric | cm/s | Mitral A Point Velocity | Mitral Inflow A Velocity |
| Mitral_E_Point_Velocity | Numeric | cm/s | Mitral E Point Velocity | Mitral Inflow E Velocity |
| Mitral_E_to_A_Ratio | Numeric | unitless | Mitral E to A Ratio | Mitral E to A Ratio |
| Mitral_Regurgitant_Volume | Numeric | cm³ | Mitral Regurgitant Volume | Mitral Regurgitant Volume |
| Mitral_Valve_Area | Numeric | cm² | Mitral Valve Area | Mitral Valve Area |
| MR_Aliasing_Velocity | Numeric | cm/s | MR Aliasing Velocity | Mitral Regurgitation Aliasing Velocity |
| MR_ERO_PISA | Numeric | cm² | MR ERO PISA | Mitral Regurgitation ERO PISA |
| MR_Flow_Convergence_Radius | Numeric | cm | MR Flow Convergence Radius | Mitral Regurgitation Flow Convergence Radius |
| MR_Flow_Rate_PISA | Numeric | cm³/s | MR Flow Rate PISA | Mitral Regurgitation Flow Rate PISA |
| MR_Mean_Gradient | Numeric | mmHg | MR Mean Gradient | Mitral Regurgitation Mean Gradient |
| MR_Mean_Velocity | Numeric | cm/s | MR Mean Velocity | Mitral Regurgitation Mean Velocity |
| MR_Orifice_PISA | Numeric | cm² | MR Orifice PISA | Mitral Regurgitation Orifice PISA |
| MR_Peak_Gradient | Numeric | mmHg | MR Peak Gradient | Mitral Regurgitation Peak Gradient |
| MR_Peak_Velocity | Numeric | cm/s | MR Peak Velocity | Mitral Regurgitation Peak Velocity |
| MR_Velocity_Time_Integral | Numeric | cm | MR Velocity Time Integral | Mitral Regurgitation Velocity Time Integral |
| MV_Area_PHT | Numeric | cm² | MV Area PHT | Mitral Valve Area PHT |
| MV_Area_Planimetry | Numeric | cm² | MV Area Planimetry | Mitral Valve Area Planimetry |
| MV_Deceleration_Slope | Numeric | cm/s² | MV Deceleration Slope | Mitral Valve Deceleration Slope |
| MV_Deceleration_Time | Numeric | ms | MV Deceleration Time | Mitral Valve Deceleration Time |
| MV_Mean_Gradient | Numeric | mmHg | MV Mean Gradient | Mitral Valve Mean Gradient |
| MV_Mean_Velocity | Numeric | cm/s | MV Mean Velocity | Mitral Valve Mean Velocity |
| MV_MPI | Numeric | unitless | MV MPI | Mitral Valve MPI |
| MV_Peak_Gradient | Numeric | mmHg | MV Peak Gradient | Mitral Valve Peak Gradient |
| MV_Peak_Velocity | Numeric | cm/s | MV Peak Velocity | Mitral Valve Peak Velocity |
| MV_PHT_Velocity | Numeric | cm/s | MV PHT Velocity | Mitral Valve PHT Velocity |
| MV_Pressure_Half_Time | Numeric | ms | MV Pressure Half Time | Mitral Valve Pressure Half Time |
| MV_Regurgitant_Fraction | Numeric | unitless | MV Regurgitant Fraction | Mitral Valve Regurgitant Fraction |
| MV_Regurgitant_Volume | Numeric | cm³ | MV Regurgitant Volume | Mitral Valve Regurgitant Volume |
| MV_Velocity_Time_Integral | Numeric | cm | MV Velocity Time Integral | Mitral Valve Velocity Time Integral |
| MVmeangradienttext | Numeric |  | MV Mean Gradient Text | Mitral Valve Mean Gradient extracted from Text data |
| MVpeakgradienttext | Numeric |  | MV Peak Gradient Text | Mitral Valve Peak Gradient extracted from Text data |
| MVpeakvelocitytext | Numeric |  | MV Peak Velocity Text | Mitral Valve Peak Velocity extracted from Text data |
| PASP_calculated | Numeric | cm/s | PASP calculated | Pulmonary Artery Systolic Pressure calculated |
| PASPtext | Numeric | mmHg | PASP Text | Pulmonary Artery Systolic Pressure extracted from Text data |
| PI_Diastolic_Velocity | Numeric | cm/s | PI Diastolic Velocity | Pulmonary Regurgitation Diastolic Velocity |
| PI_Peak_Gradient | Numeric | mmHg | PI Peak Gradient | Pulmonary Regurgitation Peak Gradient |
| PI_Peak_Velocity | Numeric | cm/s | PI Peak Velocity | Pulmonary Regurgitation Peak Velocity |
| PI_Pressure_Half_Time | Numeric | ms | PI Pressure Half Time | Pulmonary Regurgitation Pressure Half Time |
| Pulmonary_Acceleration_Time | Numeric | ms | Pulmonary Acceleration Time | Pulmonary Acceleration Time |
| Pulmonary_Vein_A_Duration | Numeric | ms | Pulmonary Vein A Duration | Pulmonary Vein A Duration |
| Pulmonary_Vein_A_to_Mitral_A_Ratio | Numeric | unitless | Pulmonary Vein A to Mitral A Ratio | Pulmonary Vein A to Mitral A Ratio |
| Pulmonary_Vein_A_Velocity | Numeric | cm/s | Pulmonary Vein A Velocity | Pulm Vein A wave Reversal Velocity |
| Pulmonary_Vein_Diastolic_Velocity | Numeric | cm/s | Pulmonary Vein Diastolic Velocity | Pulmonary Vein Diastolic Velocity |
| Pulmonary_Vein_S_D_Ratio | Numeric | unitless | Pulmonary Vein S/D Ratio | Pulmonary Vein S/D Ratio |
| Pulmonary_Vein_Systolic_Velocity | Numeric | cm/s | Pulmonary Vein Systolic Velocity | Pulmonary Vein Systolic Velocity |
| PV_Area_Cont_Eq_vti | Numeric | cm² | PV Area Cont Eq vti | Pulmonary Valve Area Cont Eq vti |
| PV_Mean_Gradient | Numeric | mmHg | PV Mean Gradient | Pulmonary Valve Mean Gradient |
| PV_Mean_Velocity | Numeric | cm/s | PV Mean Velocity | Pulmonary Valve Mean Velocity |
| PV_Peak_Gradient | Numeric | mmHg | PV Peak Gradient | Pulmonary Valve Peak Gradient |
| PV_Peak_Velocity | Numeric | cm/s | PV Peak Velocity | Pulmonary Valve Peak Velocity |
| PV_Velocity_Time_Integral | Numeric | cm | PV Velocity Time Integral | Pulmonary Valve Velocity Time Integral |
| QpQs_Shunt_Ratio | Numeric | unitless | QpQs Shunt Ratio | QpQs Shunt Ratio |
| RA_Area | Numeric | cm² | RA Area | Right Atrial Area |
| RA_Systolic_Diameter_LX | Numeric | cm | RA Systolic Diameter LX | Right Atrial Systolic Diameter Long Axis |
| RA_Systolic_Diameter_Transverse | Numeric | cm | RA Systolic Diameter Transverse | Right Atrial Systolic Diameter Transverse |
| RApressuretext | Numeric |  | RA Pressure Text | Right Atrial Pressure |
| RAVItext | Numeric |  | RAVI Text | Right Atrial Volume Index Final |
| RAVtext | Numeric |  | RAV Text | Right Atrial Volume Index |
| Rhythm | Numeric | unitless | Rhythm | ECG rhythm |
| Right_Atrial_Pressure | Numeric | mmHg | Right Atrial Pressure | Right Atrial Pressure |
| Right_Atrial_Pressure_Hierarchy | Numeric | mmHg | Right Atrial Pressure Hierarchy | Right Atrial Pressure - Based on hierarchy (RApressuretext, Right Atrial Pressure) |
| Right_Atrial_Volume | Numeric | ml | Right Atrial Volume | Right Atrial Volume |
| Right_Atrial_Volume_Index | Numeric |  | Right Atrial Volume Index | Right Atrial Volume Index - Based on heirarchy (RAVItext, Right Atrial Volume Index) |
| Right_Atrial_Volume_Index_Hierarchy | Numeric |  | Right Atrial Volume Index Hierarchy | Right Atrial Volume Index - Based on hierarchy (RAVItext, Right Atrial Volume Index) |
| Right_Atrial_Volume_Hierarchy | Numeric | ml | Right Atrial Volume Hierarchy | Right Atrial Volume - Based on hierarchy (RAVtext, Right Atrial Volume) |
| Right_Ventricular_Systolic_Pressure | Numeric | mmHg | Right Ventricular Systolic Pressure | Right Ventricular Systolic Pressure estimated from Tricuspid Regurgitation velocity |
| RV_Diameter | Numeric | cm | RV Diameter | Right Ventricular Diameter |
| RV_Diastolic_Area_4C | Numeric | cm² | RV Diastolic Area 4C | Right Ventricular Diastolic Area Measured from Apical 4-chamber view |
| RV_Diastolic_Basal_Diameter | Numeric | cm | RV Diastolic Basal Diameter | Right Ventricular Diastolic Basal Diameter |
| RV_Diastolic_Mid_Diameter | Numeric | cm | RV Diastolic Mid Diameter | Right Ventricular Diastolic Mid Diameter |
| RV_Fractional_Area_Change | Numeric | unitless | RV Fractional Area Change | Right Ventricular Fractional Area Change |
| RV_Internal_Dim_ED_PLAX | Numeric | cm | RV Internal Dim ED PLAX | Right Ventricular Internal Dimension at end diastole measured from PLAX view |
| RV_Myoc_Perform_Index | Numeric | unitless | RV Myoc Perform Index | Right Ventricular Myocardial Performancd Index (MPI) |
| RV_Systolic_Area_4C | Numeric | cm² | RV Systolic Area | Right Ventricular Systolic Area Measured from Apical 4-chamber view |
| RV_Systolic_Diameter_MM | Numeric | cm | RV Systolic Diameter MM | Right Ventricular Systolic Diameter M-Mode |
| RV_Wall_Diastolic_Diameter_MM | Numeric | cm | RV Wall Diastolic Diameter MM | Right Ventricular Wall Diastolic Diameter M-Mode |
| RV_Wall_Diastolic_Thickness | Numeric | cm | RV Wall Diastolic Thickness | Right Ventricular Wall Diastolic Thickness |
| RV_Wall_Systolic_Diameter_MM | Numeric | cm | RV Wall Systolic Diameter MM | Right Ventricular Wall Systolic Diameter M-Mode |
| RV_Wall_Systolic_Thickness | Numeric | cm | RV Wall Systolic Thickness | Right Ventricular Wall Systolic Thickness |
| RVOT_Diameter | Numeric | cm | RVOT Diameter | Right Ventricular Outflow Tract Diameter |
| RVOT_Ejection_Time | Numeric | s | RVOT Ejection Time | Right Ventricular Outflow Tract Ejection Time |
| RVOT_Mean_Gradient | Numeric | mmHg | RVOT Mean Gradient | Right Ventricular Outflow Tract Mean Gradient |
| RVOT_Mean_Velocity | Numeric | cm/s | RVOT Mean Velocity | Right Ventricular Outflow Tract Mean Velocity |
| RVOT_Peak_Gradient | Numeric | mmHg | RVOT Peak Gradient | Right Ventricular Outflow Tract Peak Gradient |
| RVOT_Peak_Velocity | Numeric | cm/s | RVOT Peak Velocity | Right Ventricular Outflow Tract Peak Velocity |
| RVOT_Velocity_Time_Integral | Numeric | cm | RVOT Velocity Time Integral | Right Ventricular Outflow Tract Velocity Time Integral |
| Systolic_BP | Numeric | mmHg | Systolic BP | Systolic BP |
| Thoracic_Aorta_Diameter | Numeric | cm | Thoracic Aorta Diameter | Thoracic Aorta Diameter |
| TR_Mean_Gradient | Numeric | mmHg | TR Mean Gradient | Tricuspid Regurgitation Mean Gradient |
| TR_Mean_Velocity | Numeric | cm/s | TR Mean Velocity | Tricuspid Regurgitation Mean Velocity |
| TR_Peak_Gradient | Numeric | mmHg | TR Peak Gradient | Tricuspid Regurgitation Peak Gradient |
| TR_Peak_Velocity | Numeric | cm/s | TR Peak Velocity | Tricuspid Regurgitation Peak Velocity |
| RVSP_Combined | Numeric |  | Estimated RVSP assuming RAP = 5 including text extraction |  |
| TR_Velocity_Time_Integral | Numeric | cm | TR Velocity Time Integral | Tricuspid Regurgitation Velocity Time Integral |
| Tricuspid_Annular_Plane_Systolic_Excursion_M_mode | Numeric | cm | Tricuspid Annular Plane Systolic Excursion M mode | Tricuspid Annular Plane Systolic Excursion M mode |
| Tricuspid_Annular_Plane_Systolic_Excursion_TDI | Numeric | cm/s | Tricuspid Annular Plane Systolic Excursion TDI | Tricuspid Annular Plane Systolic Excursion TDI |
| TV_E_to_A_Ratio | Numeric | unitless | TV E to A Ratio | Tricuspid Valve Diastolic Doppler E to A Ratio |
| TV_Mean_Gradient | Numeric | mmHg | TV Mean Gradient | Tricuspid Valve Diastolic Doppler Mean Gradient |
| TV_Mean_Velocity | Numeric | cm/s | TV Mean Velocity | Tricuspid Valve Diastolic Doppler Mean Velocity |
| TV_Peak_A_Velocity | Numeric | cm/s | TV Peak A Velocity | Tricuspid Valve Diastolic Doppler Peak A Velocity |
| TV_Peak_E_Velocity | Numeric | cm/s | TV Peak E Velocity | Tricuspid Valve Diastolic Doppler Peak E Velocity |
| TV_Peak_Gradient | Numeric | mmHg | TV Peak Gradient | Tricuspid Valve Diastolic Doppler Peak Gradient |
| TV_Peak_Velocity | Numeric | cm/s | TV Peak Velocity | Tricuspid Valve Diastolic Doppler Peak Velocity |
| TV_Velocity_Time_Integral | Numeric | cm | TV Velocity Time Integral | Tricuspid Valve Diastolic Doppler Velocity Time Integral |
| TVmeangradienttext | Numeric |  | TV Mean Gradient Text | Tricuspid Valve Diastolic Doppler Mean Gradient from Text Extraction |
| TVpeakgradienttext | Numeric |  | TV Peak Gradient Text | Tricuspid Valve Diastolic Doppler Peak Gradient from Text Extraction |
| TVpeakvelocitytext | Numeric |  | TV Peak Velocity Text | Tricuspid Valve Diastolic Doppler Peak Velocity from Text Extraction |
| MV_Lateral_E_Prime_Velocity | Numeric |  | MV Lateral E Prime Velocity |  |
| MV_Septal_E_Prime_Velocity | Numeric |  | MV Septal E Prime Velocity |  |
| LVOT_Stroke_Volume_Index | Numeric |  | LVOT Stroke Volume Index |  |
| Stroke_Volume_Index | Numeric |  | Stroke Volume Index (ml/m2) |  |
| sinus_rhythm | Numeric |  | Sinus Rhythm determined by mitral inflow |  |
| af_or_other_atrial_arrhythmia | Numeric |  | AF or other Atrial Arrhythmia determined by mitral inflow |  |
| undetermined_rhythm | Numeric |  | Undetermined rhythm based on mitral inflow |  |
| eRVSP_from_TR | Numeric |  | eRVSP from TR velocity assuming RAP=5mmHg |  |
| LVEF_MM_Teich | Numeric |  |  |  |
| LVEF_2D_Teich | Numeric |  |  |  |

**Legend:** Shown are the variable names, data type, unit names, labels, and descriptors for a standard list of 280 echocardiographic variables used to train the derivation model (reproduced with permission from Strange G, *et al. Open Heart* 2023;10:e002265.)

**Supplementary Table 2:** Comparison of Internal Validation Cohort and External Validation Cohort

|  | **Internal Validation Cohort**  **(N=184,301)** | **External Validation Cohort (N=31,141)** |
| --- | --- | --- |
| Age, years | 61.8 ± 17.8 | 77.5 ± 8.3 |
| Women, % | 87,987 (47.7%) | 16,230 (52.1%) |
| Body mass index, kg/m^2^ | 28.1 ± 6.4 | 27.5 ± 6.1 |
| Systolic/Diastolic BP, mmHg | 134 ± 22 / 77 ± 12 | 131 ± 22 / 69 ± 13 |
| Heart rate, bpm | 72.4 ± 15.5 | 73.8 ± 15.9 |
| Peak velocity, m/s | 1.4 (1.2-1.7) | 1.7 ± 0.7 |
| Mean gradient, mmHg | 4.5 (3.1-7.6) | 20.1 ± 14.5 |
| Aortic valve area, cm^2^ | 2.5 (1.9-3.2) | 1.4 ± 0.6 |
| Peak TR velocity, m/s | 2.6 ± 0.5 | 2.8 ± 0.5 |
| Left atrial volume index, mL/m^2^ | 41.6 ± 28.8 | 30.8 ± 11.4 |
| LV end-diastolic dimension, cm | 4.7 ± 0.7 | 4.5 ± 0.8 |
| LV end-systolic dimension, cm | 3.0 ± 0.8 | 2.8 ± 0.8 |
| LV ejection fraction, % | 62 ± 13 | 62 ± 17 |
| Transmitral E/e’ ratio | 10.6 ± 5.0 | 12.3 ± 5.6 |
| Transmitral E/A ratio | 1.2 ± 0.7 | 1.1 ± 0.7 |
| Stroke volume index, mL/m^2^ | 40.1 ± 11.7 | 39.1 ± 11.9 |

**Legend:** Displayed is a comparison of the baseline characteristics of the cohorts used for internal and external validation across features shared between the two cohorts. Values are presented in means and standard deviations unless otherwise indicated. Values in parentheses indicate the interquartile range. BP = blood pressure, LV = left ventricular, TR = tricuspid regurgitation.

**Supplementary Table 3:** Baseline characteristics by Group and Receipt of Aortic Valve Replacement

|  | **Severe AS Groups (N=2,012)** | | | | | |
| --- | --- | --- | --- | --- | --- | --- |
|  | **Group 1**  **AVR**  **(N=340)** | **Group 1**  **no AVR**  **(N=1,209)** | **p-value** | **Group 2 AVR**  **(N=20)** | **Group 2**  **no AVR (N=443)** | **p-value** |
| Age, years | 79.1 ± 6.5 | 84.1 ± 8.2 | < 0.001 | 78.9 ± 5.5 | 84.0 ± 7.9 | < 0.001 |
| Women, % | 153 (45.0) | 671 (55.5) | < 0.001 | < 11 | 305 (68.9) | 0.09 |
| White, % | 317 (93.2) | 1111 (91.9) | 0.05 | 20 (100) | 387 (87.4) | 0.24 |
| Black, % | < 11 | 51 (4.2) | 0.05 | 0 (0.0) | 30 (6.8) | 0.24 |
| Other, % | 17 (5.0) | 47 (3.9) | 0.05 | 0 (0.0) | 26 (5.9) | 0.24 |
| Inpatient at Echo, % | 192 (56.5) | 893 (73.9) | < 0.001 | 12 (60.0) | 334 (75.4) | 0.18 |
| Body mass index, kg/m^2^ | 28.1 ± 5.6 | 25.9 ± 5.4 | < 0.001 | 26.0 ± 4.3 | 25.6 ± 5.9 | 0.76 |
| Systolic / Diastolic BP, mmHg | 132 ± 21 / 69 ± 12 | 126 ± 23 / 66 ± 14 | <0.001 | 127 ± 19 / 71 ± 11 | 129 ± 23 / 65 ± 14 | 0.75 / 0.02 |
| Heart rate, bpm | 69.8 ± 13.3 | 75.7 ± 15.7 | < 0.001 | 72.4 ± 12.7 | 74.1 ± 15.8 | 0.57 |
| eGFR, L/min/1.73m^2^ | 127.5 ± 155.6 | 113.9 ± 135.7 | 0.25 | 82.7 ± 126.5 | 128.5 ± 121.0 | 0.29 |
| NT-proBNP, pg/ml | 6,074 ± 7,035 | 12,475 ± 14,672 | < 0.001 | 9,510 ± 5,374 | 10,918 ± 11,004 | 0.70 |
| Type II Diabetes, % | 116 (34.1) | 409 (33.8) | 0.95 | < 11 | 155 (35.0) | 0.23 |
| Hypertension, % | 270 (79.4) | 913 (75.5) | 0.15 | 15 (75.0) | 336 (75.9) | > 0.99 |
| Coronary artery disease, % | 254 (74.7) | 833 (68.9) | 0.04 | 14 (70.0) | 305 (68.9) | > 0.99 |
| Heart failure, % | 199 (58.5) | 821 (67.9) | 0.0013 | < 11 | 306 (69.1) | 0.03 |
| Peak velocity, m/s | 4.1 ± 0.7 | 3.8 ± 0.8 | < 0.001 | 3.3 ± 0.4 | 2.8 ± 0.6 | < 0.001 |
| Mean gradient, mmHg | 41.5 ± 15.1 | 35.0 ± 16.4 | < 0.001 | 25.3 ± 5.5 | 19.7 ± 7.3 | < 0.001 |
| Aortic valve area, cm^2^ | 0.79 ± 0.19 | 0.81 ± 0.19 | 0.03 | 1.13 ± 0.05 | 1.28 ± 0.24 | < 0.001 |
| $\geq$ Moderate aortic regurgitation | < 11 | 35 (2.9) | 0.18 | < 11 | 11 (2.5) | 0.42 |
| Peak TR velocity, m/s | 2.8 ± 0.5 | 3.1 ± 0.5 | < 0.001 | 2.9 ± 0.5 | 3.1 ± 0.5 | 0.29 |
| ≥ Moderate TR | 35 (10.3) | 322 (26.6) | < 0.001 | 0 (0.0) | 138 (31.2) | < 0.001 |
| Left atrial volume index, mL/m^2^ | 32.3 ± 10.9 | 40.7 ± 12.5 | < 0.001 | 35.9 ± 14.9 | 37.3 ± 13.9 | 0.92 |
| LV end-diastolic dimension, cm | 4.5 ± 0.7 | 4.5 ± 0.8 | 0.40 | 4.4 ± 0.8 | 4.4 ± 0.9 | 0.90 |
| LV end-systolic dimension, cm | 2.8 ± 0.8 | 2.9 ± 0.9 | 0.14 | 3.0 ± 0.9 | 3.0 ± 1.0 | 0.86 |
| LV ejection fraction, % | 61.8 ± 16.8 | 57.9 ± 19.7 | < 0.001 | 56.1 ± 19.3 | 52.1 ± 19.4 | 0.37 |
| Transmitral E/e’ ratio | 15.1 ± 6.4 | 18.1 ± 8.9 | < 0.001 | 13.3 ± 4.1 | 16.9 ± 7.4 | 0.12 |
| Transmitral E/A ratio | 1.1 ± 0.6 | 1.3 ± 0.8 | 0.01 | 1.1 ± 0.5 | 1.3 ± 0.8 | 0.20 |
| Stroke volume index, mL/m^2^ | 38.1 ± 11.2 | 36.9 ± 12.8 | 0.26 | 45.3 ± 9.2 | 34.9 ± 13.6 | 0.04 |
| ≥ Moderate Mitral Regurgitation, % | 62 (18.2) | 341 (28.2) | < 0.001 | < 11 | 139 (31.4) | 0.14 |
| Anticoagulant, % | 50 (14.7) | 184 (15.2) | 0.86 | < 11 | 68 (15.4) | 0.33 |
| Diuretic, % | 102 (30.0) | 416 (34.44) | 0.13 | < 11 | 170 (38.4) | 0.04 |
| Neurohormonal antagonists, % | 116 (34.1) | 341 (28.2) | 0.04 | < 11 | 137 (30.9) | 0.81 |
| Anti-platelet, % | 143 (42.1) | 429 (35.5) | 0.03 | < 11 | 175 (39.5) | 0.49 |
| Anti-arrhythmic, % | 130 (38.2) | 489 (40.5) | 0.49 | < 11 | 197 (44.5) | 0.49 |
| Beta-blocker, % | 126 (37.1) | 491 (40.6) | 0.26 | < 11 | 196 (44.2) | 0.82 |
| PCI, % | < 11 | 62 (5.1) | 0.06 | 0 (0.0) | 29 (6.6) | 0.63 |
| CABG, % | 56 (16.5) | < 11 | < 0.001 | < 11 | 13 (2.9) | 0.004 |

**Legend**: This table shows the demographic, clinical, echocardiographic profile, and treatment profile of groups 1 and 2 identified by the AI-DSA stratified by receipt or non-receipt of aortic valve replacement (AVR). Values are presented as means **±** standard deviations or counts (percentages). The number of individuals with complete data for each variable are indicated. Group 1 indicates individuals above the F1 threshold meeting guidelines for severe AS. Group 2 indicates individuals above the F1 threshold who did not meet guideline criteria for severe AS. CABG = coronary artery bypass grafting, eGFR = estimated glomerular filtration rate, LV = left ventricular, N = number of patients, NT-proBNP = N-terminal pro-brain natriuretic peptide, PCI = percutaneous coronary intervention, TR = tricuspid regurgitation. Cell numbers under 11 are suppressed by Medicare data use policy.

**Supplementary Table 4:** Rates and Timing of Receipt and Sub-type of Aortic Valve Replacement at 1-year Following Echocardiography

| **Group** | **AVR**  **at 1-year**  **(N=325)** | **Median (IQR) Time to AVR** | **TAVR at 1-year**  **(N = 35)** | **Median (IQR) Time to TAVR** | **SAVR**  **at 1-year**  **(N = 291)** | **Median (IQR) Time to SAVR** |
| --- | --- | --- | --- | --- | --- | --- |
| 1 (N=1,549) | 256  (16.5%) | 31.0  (5.0-92.0) | 33 (2.1%) | 144.0  (83.0-191.0) | 224  (14.5%) | 16.5  (4.0-73.5) |
| 2 (N= 63) | 11  (2.4%) | 10.0  (4.0-168.0) | 0  (0.0%) | N/A | 11  (2.4%) | 10.0  (4.0-168.0) |
| 3 (N= 79) | 13  (1.3%) | 57.0  (6.0-135.0) | < 11 | 2.0  (2.0-2.0) | 12  (1.2%) | 59.5  (6.5-175.5) |
| 4 (N=28,150) | 45  (0.2%) | 18.0  (6.0-82.0) | < 11 | 341.0  (341.0-341.0) | 44  (0.2%) | 17.5  (6.0-79.0) |

**Legend**: This table shows the number, timing, and type of aortic valve replacement (AVR) at 1-year after the index echocardiogram by according to predicted AI-DSA group. Overall AVRs are presented as well as stratified by surgical (SAVR) or transcatheter aortic valve receipt (TAVR). Group 1 indicates individuals above the F1 threshold meeting guidelines for severe AS. Group 2 indicates individuals above the F1 threshold who did not meet guideline criteria for severe AS. Group 3 indicates those below the F1 threshold in the moderate aortic valve severity category. Group 4 indicates those below the F1 threshold in the mild or less aortic valve severity category. AI-DSA = artificial intelligence decision support algorithm, AS = aortic stenosis, IQR = interquartile range, N/A = not applicable. Cell numbers under 11 are suppressed by Medicare data use policy.

**Supplementary Table 5:** Baseline Characteristics of Discordant Groups

|  | **Group A**  **(N=978)** | **Group B**  **(N=1,034)** | **Group D**  **(N=29,124)** |
| --- | --- | --- | --- |
| Age, years | 83.6 ± 7.9 | 82.8 ± 8.2 | 77.0 ± 8.2 |
| Women, % | 530 (54.2%) | 609 (58.9%) | 15,091 (51.8%) |
| White, % | 906 (92.6%) | 929 (89.9%) | 23,747 (81.5%) |
| Black, % | 35 (3.6%) | 52 (5.0%) | 2,723 (9.4%) |
| Other, % | 37 (3.8%) | 53 (5.1%) | 2,654 (9.1%) |
| Inpatient at Echo, % | 707 (72.3%) | 724 (70.0%) | 17,068 (58.6%) |
| Body mass index, kg/m^2^ | 26.3 ± 5.5 | 26.3 ± 5.6 | 27.6 ± 6.1 |
| Systolic / Diastolic BP, mmHg | 125 ± 22 /  65 ± 14 | 130 ± 23 /  66 ± 14 | 131 ± 22 /  70 ± 13 |
| Heart rate, bpm | 74.7 ± 15.7 | 74.0 ± 15.2 | 73.7 ± 15.9 |
| eGFR, L/min/1.73m^2^ | 115.9 ± 136.3 | 121.5 ± 134.3 | 147.6 ± 181.0 |
| NT-proBNP, pg/ml | 12,572 ± 14,586 | 10,249 ± 11,753 | 6,407 ± 9,720 |
| Type II Diabetes, % | 342 (35.0%) | 342 (33.1%) | 8,678 (29.8%) |
| Hypertension, % | 746 (76.3%) | 788 (76.2%) | 18,538 (63.7%) |
| Coronary artery disease, % | 695 (71.1%) | 711 (68.8%) | 13,860 (47.6%) |
| Heart failure, % | 666 (68.1%) | 669 (64.7%) | 11,552 (39.7%) |
| Peak velocity, m/s | 4.1 ± 0.8 | 3.1 ± 0.7 | 1.6 ± 0.5 |
| Mean gradient, mmHg | 41.9 ± 16.2 | 24.1 ± 10.6 | 12.9 ± 6.0 |
| Aortic valve area, cm^2^ | 0.72 ± 0.16 | 1.03 ± 0.21 | 1.75 ± 0.57 |
| $\geq$ Moderate aortic regurgitation | 31 (3.2%) | 21 (2.0%) | 230 (0.8%) |
| Peak TR velocity, m/s | 3.1 ± 0.5 | 3.0 ± 0.5 | 2.7 ± 0.5 |
| ≥ Moderate TR | 243 (24.9%) | 252 (24.4%) | 3,614 (12.4%) |
| Left atrial volume index, mL/m^2^ | 39.8 ± 13.2 | 35.9 ± 11.4 | 30.4 ± 11.2 |
| LV end-diastolic dimension, cm | 4.5 ± 0.8 | 4.4 ± 0.9 | 4.5 ± 0.8 |
| LV end-systolic dimension, cm | 3.0 ± 0.9 | 2.9 ± 1.0 | 2.8 ± 0.8 |
| LV ejection fraction, % | 57.7 ± 19.7 | 56.9 ± 19.2 | 62.0 ± 16.4 |
| Transmitral E/e’ ratio | 17.7 ± 8.5 | 16.7 ± 7.8 | 12.0 ± 5.2 |
| Transmitral E/A ratio | 1.3 ± 0.8 | 1.2 ± 0.7 | 1.1 ± 0.7 |
| Stroke volume index, mL/m^2^ | 37.1 ± 12.1 | 37.1 ± 13.3 | 39.2 ± 11.8 |
| ≥ Mod. Mitral Regurgitation, % | 281 (28.7%) | 264 (25.5%) | 2,845 (9.8%) |
| Anticoagulant, % | 159 (16.3%) | 144 (13.9%) | 3,807 (13.1%) |
| Diuretic, % | 352 (36.0%) | 339 (32.8%) | 8,578 (29.5%) |
| Neurohormonal antagonists, % | 286 (29.2%) | 315 (30.5%) | 9,447 (32.4%) |
| Anti-platelet, % | 378 (38.7%) | 375 (36.3%) | 10,790 (37.1%) |
| Anti-arrhythmic, % | 400 (40.9%) | 423 (40.9%) | 11,445 (39.3%) |
| Beta-blocker, % | 403 (41.2%) | 418 (40.4%) | 12,045 (41.4%) |
| PCI, % | 39 (4.0%) | 61 (5.9%) | 1,366 (4.7%) |
| CABG, % | 44 (4.5%) | 31 (3.0%) | 723 (2.5%) |

**Legend:** This table shows the demographic, clinical, echocardiographic profile, and treatment profile of groups according to their categorization by the AI-DSA and clinical grading. Values are presented as means **±** standard deviations or counts (percentages). Group A identifies individuals determined to have the severe AS phenotype by both the AI-DSA and clinical interpretation. Group B identifies individuals determined to have the severe AS phenotype by the AI-DSA but non-severe AS by clinical interpretation. Group D identifies individuals determined to have non-severe AS by both the AI-DSA and clinical interpretation. CABG = coronary artery bypass grafting, eGFR = estimated glomerular filtration rate, LV = left ventricular, N = number of patients, NT-proBNP = N-terminal pro-brain natriuretic peptide, PCI = percutaneous coronary intervention, TR = tricuspid regurgitation.

**Supplemental Table 6:** Rates of Aortic Valve Replacement at 1-year and Mortality at 5-years after Echocardiography According to Artificial Intelligence Agreement with Clinical Interpretation

| **Group** | **AVR**  **at 1-year**  **(N= 325)** | **Death**  **at 5-years**  **(N = 14,482)** | **Median (IQR) Time to Death (years)** | **Unadjusted HR (95% CI) of**  **5-year Death** | **p-value** |
| --- | --- | --- | --- | --- | --- |
| A (N=978) | 199 (20.4%) | 742 (75.9%) | 0.4 (0.1-1.6) | 2.48 (2.30-2.67) | < 0.001 |
| B (N=1,034) | 68 (6.6%) | 760 (73.5%) | 0.6 (0.1-2.0) | 2.21 (2.06-2.38) | < 0.001 |
| C (N <11) | 0 (0%) | < 11 | 0.05 (0.04-0.07) | 1.03 (0.26-4.11) | 0.97 |
| D (N=29,124) | 58 (0.2%) | 12,978 (44.6%) | 0.9 (0.2-2.5) | Ref | Ref |

**Legend:** This table shows the rate of aortic valve replacement (AVR) at 1-year after the index echocardiogram, rates of all-cause mortality at 5-years, median time to mortality within 5-years (in years), and crude hazard ratios (HRs) and 95% confidence intervals (CIs) for all-cause mortality by clinical interpretation group. Group A consists of individuals for whom clinical interpretation and the AI-DSA agreed on severe AS status. Group B represents individuals categorized by the AI-DSA as having severe AS but not by clinical interpretation. Group C represents individuals categorized by clinical interpretation as having severe AS but not by AI-DSA. Group D represents those individuals for whom AI-DSA and clinical interpretation agreed that there was non-severe AS. AI-DSA = artificial intelligence decision support algorithm, AS = aortic stenosis, IQR = interquartile range, N = number of individuals, Ref = reference category. Cell numbers under 11 are suppressed by Medicare data use policy.

**Supplemental Table 7:** Variable Importance List from Random Forest Model Evaluating Variables Associated with the Artificial Intelligence Algorithm Predicted Probability

| **Term** | **Number of Splits** | **Portion** |
| --- | --- | --- |
| Age | 4186 | 0.2006 |
| TR velocity | 4179 | 0.1088 |
| MR Severity | 591 | 0.0682 |
| Systolic BP | 3676 | 0.0494 |
| Diastolic BP | 3848 | 0.0484 |
| Left ventricular EF | 3781 | 0.0482 |
| Stroke volume index | 1935 | 0.0391 |
| Glomerular filtration rate | 2918 | 0.0386 |
| LV end-diastolic diameter | 4545 | 0.0342 |
| Body mass index | 3844 | 0.0324 |
| E/e’ | 1850 | 0.0312 |
| Heart failure | 817 | 0.0264 |
| Heart rate | 3386 | 0.0263 |
| LV end-systolic diameter | 3025 | 0.0250 |
| Coronary artery disease | 1234 | 0.0171 |
| NT-proBNP | 762 | 0.0164 |
| E/A ratio | 1537 | 0.0164 |
| Gender | 3240 | 0.0147 |
| Atrial fibrillation | 1134 | 0.0108 |
| Osteoarthritis | 1486 | 0.0092 |
| Anemia | 1601 | 0.0091 |
| Chronic kidney disease | 1329 | 0.0084 |
| Hyperlipidemia | 1733 | 0.0081 |
| Beta blocker | 1576 | 0.0078 |
| TR severity | 572 | 0.0073 |
| Diabetes | 1540 | 0.0072 |
| Antiplatelet use | 1843 | 0.0069 |
| Depression | 944 | 0.0069 |
| Anti-arrhythmic drug | 1445 | 0.0067 |
| Diuretics | 1350 | 0.0067 |
| COPD | 776 | 0.0057 |
| Neurohumoral agents | 1535 | 0.0054 |
| Dementia | 690 | 0.0053 |
| Inpatient/outpatient status | 1812 | 0.0052 |
| Race | 625 | 0.0040 |
| Hypothyroidism | 441 | 0.0039 |
| Osteoporosis | 317 | 0.0038 |
| Hypertension | 1445 | 0.0035 |
| LAVI | 277 | 0.0034 |
| Cataracts | 616 | 0.0032 |
| CABG | 192 | 0.0023 |
| History of AMI | 322 | 0.0022 |
| AR severity | 34 | 0.0021 |
| Anticoagulants | 346 | 0.0021 |
| Glaucoma | 227 | 0.0020 |
| Benign prostatic hypertrophy | 264 | 0.0019 |
| History of stroke/TIA | 350 | 0.0019 |
| Alzheimer’s dementia | 164 | 0.0017 |
| Asthma | 184 | 0.0016 |
| Breast cancer | 59 | 0.0006 |
| Colorectal cancer | 80 | 0.0005 |
| Prostate cancer | 72 | 0.0004 |
| PCI | 110 | 0.0004 |
| Lung cancer | 40 | 0.0003 |
| Hip fracture | 16 | 0.0002 |
| Endometrial cancer | 0 | 0.0000 |

**LEGEND:** Shown are results of a random forest model incorporating the AI model’s predicted probability of AS as the response variable and all baseline characteristics (excluding aortic valve parameters) as predictors. Variables are listed in the order of importance according to the number of tree splits that include a given variable and the proportion of the total number of splits that include the variable. Thus, those variables at the top of the list are the variables most associated with the AI’s prediction of an individual’s AS status.

**Supplementary Figure 1.** Kaplan Meier Curve Demonstrating Time to All-Cause Mortality by Receipt or Non-receipt of Aortic Valve Replacement


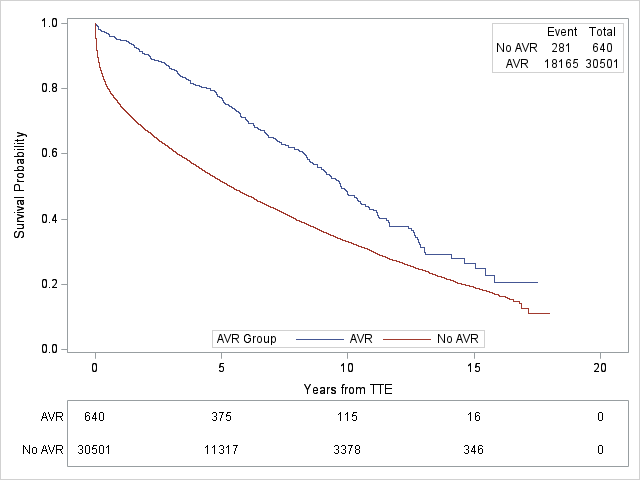


**Legend:** Displayed is a Kaplan Meier curve demonstrating time from the index echocardiogram to all-cause mortality stratified by receipt (blue line) or non-receipt (red line) of aortic valve replacement (AVR). Numbers in the risk set at 5-year increments are provided below. The log-rank p-value for comparison across groups was <0.001. TTE = transthoracic echocardiogram.

**Supplementary Figure 2.** Kaplan Meier Curve Demonstrating Time to All-Cause Mortality by Artificial Intelligence Algorithm Grouping in Outpatients


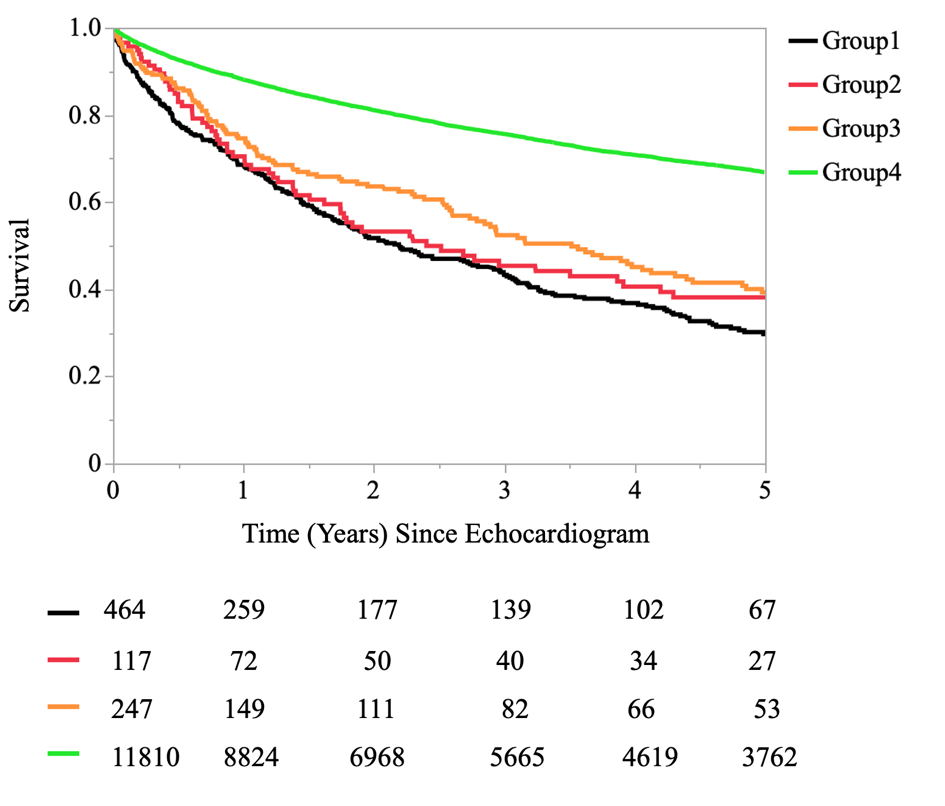


**Legend:** Displayed is a Kaplan Meier curve demonstrating time from the index echocardiogram to all-cause mortality within 5-years by AI-DSA group amongst individuals receipting echocardiography as an outpatient. Numbers in the risk set at one-year time points are provided below. Group 1 (black line) indicates individuals above the F1 threshold meeting guidelines for severe AS. Group 2 (red line) indicates individuals above the F1 threshold who did not meet guideline criteria for severe AS. Group 3 (orange line) indicates those below the F1 threshold in the moderate aortic valve severity category. Group 4 (green line) indicates those below the F1 threshold in the mild or less aortic valve severity category. The log-rank p-value for comparison across groups was <0.001. AI-DSA = artificial intelligence decision support algorithm.
